# Public‐Facing Short Videos on Paediatric General Anaesthesia and Child Neurodevelopment: Quality, Misinformation and Implications for Parent‐Centred Risk Communication

**DOI:** 10.1111/hex.70826

**Published:** 2026-08-17

**Authors:** Chong Xu, Yuchang Fang, Lei Yao, Jie Song

**Affiliations:** ^1^ Department of Anesthesiology, Nantong First People's Hospital Southeast University Nantong Jiangsu China

**Keywords:** caregiver decision‐making, health information quality, misinformation, paediatric anaesthesia, parent‐centred care, short‐video platforms

## Abstract

**Background:**

Caregivers may encounter short videos about paediatric general anaesthesia and neurodevelopment before consultation. We assessed their quality, reliability, usability and misinformation patterns, and considered the implications for parent‐centred risk communication.

**Methods:**

On 15 August 2025, we used newly created accounts to retrieve the first 100 results from Douyin and Bilibili for two Chinese search terms. Two anaesthesiologists independently assessed eligible videos using GQS, MQ‐VET, JAMA benchmarks, mDISCERN and PEMAT‐A/V. A pre‐search PPIE consultation, conducted through brief one‐to‐one discussions with 10 family contributors, informed study framing and parent‐facing interpretation.

**Results:**

Of 200 retrieved videos, 95 were included (Douyin, *n* = 74; Bilibili, *n* = 21). Douyin videos attracted more engagement, but quality did not differ by platform. Videos uploaded by anaesthesiologists, hospitals and news agencies tended to score higher than those uploaded by non‐anaesthesiologists in several domains, although these subgroup comparisons were exploratory. Misinformation occurred in 51 videos (53.7%); false safety assurances were most common (43/95, 45.3%). After false‐discovery‐rate correction, stratified analyses showed no significant differences by platform or uploader. Engagement was not a reliable indicator of quality, whereas video duration was positively correlated with mDISCERN, GQS and MQ‐VET.

**Conclusions:**

Short videos were readily accessible but uneven in reliability and transparency. Popularity did not indicate quality. Preoperative communication should address online information, correct binary claims, explain exposure‐related modifiers and direct families to trustworthy clinician‐produced resources. Caregiver exposure and decision outcomes were not measured.

**Patient or Public Contribution:**

Ten family contributors took part in brief one‐to‐one PPIE consultations during pre‐anaesthesia assessment. Their advice supported the use of lay search language and the parent‐facing interpretation of omissions and false reassurance. Contributors did not review or score videos, and their views were not treated as individual‐level research data.

## 1 Introduction

Paediatric general anaesthesia is essential to modern perioperative care, but its possible neurodevelopmental consequences remain a persistent concern for caregivers and clinicians. Early childhood is a sensitive period for brain development, and parents often ask whether anaesthetic exposure can affect later cognition, memory, learning or behaviour. The evidence is nuanced rather than categorical. The U.S. Food and Drug Administration warned that repeated or prolonged exposure in children younger than 3 years may affect the developing brain, while also noting that a single brief exposure is unlikely to cause measurable long‐term harm [1]. The PANDA and GAS studies did not identify significant deficits in global cognitive outcomes after a single short exposure. By contrast, the MASK study and later meta‐analytic evidence suggest that multiple or prolonged exposures may be associated with more specific differences, particularly in behaviour and executive function, rather than a uniform decline in intelligence [2, 3, 4, 5, 6]. Counselling should therefore focus not on whether all paediatric anaesthetic exposure is harmful, but on how age, exposure duration, cumulative exposure, surgical need and clinical context should be discussed with families.

Caregivers often encounter this question before they reach the anaesthesia clinic. They may search online or consult hospital websites, relatives, parent groups and short‐video platforms before meeting an anaesthesiologist. The World Health Organization has recognised the growing role of short videos in public health communication and the need for more science‐based health information on TikTok [7]. These formats can make complex topics easier to access, especially when written consent materials are difficult to interpret. However, algorithmic ranking, rapid production and limited source disclosure can make it hard to distinguish clinically grounded explanations from incomplete or promotional content. Studies of short‐form medical videos across other clinical topics have reported inconsistent quality, incomplete sourcing and variable reliability [8, 9, 10].

Short‐video search environments warrant separate assessment because they combine concise audiovisual presentation with algorithmic ranking, visible popularity cues and rapid recirculation. These features make information easy to encounter but can blur the distinction between professional explanation, personal opinion and promotional content. A brief video may also leave viewers with a simple conclusion after important qualifications have been omitted. Findings from other clinical topics may not transfer directly to paediatric anaesthesia, where uncertainty, procedural necessity and parental responsibility interact. Evaluation therefore needs to consider not only factual accuracy but also transparency, understandability, actionability and the ways in which risk is simplified.

From a person‐centred care perspective, meaningful participation requires more than access to information; the information must also be reliable, transparent, understandable and actionable [11]. Paediatric anaesthesia is particularly vulnerable to binary messages. Some videos imply inevitable brain injury, whereas others describe anaesthesia as harmless in all circumstances. Either framing can undermine informed discussion. Parent‐facing communication should distinguish a single brief exposure from repeated or prolonged exposure, separate animal or mechanistic evidence from human findings and explain why delaying an indicated procedure may itself cause harm.

Little is known about the quality of short videos that address the cognitive or neurodevelopmental effects of paediatric general anaesthesia. We conducted a cross‐sectional study of Douyin and Bilibili videos to evaluate quality, reliability, transparency, understandability, actionability and misinformation patterns. We also incorporated patient and public involvement and engagement (PPIE) through a one‐off consultation comprising brief one‐to‐one discussions with family contributors to inform study framing, lay terminology and parent‐facing interpretation. In addition to assessing video quality, we considered the implications of the observed content patterns for preoperative questions and caregiver participation in perioperative communication.

## 2 Materials and Methods

### 2.1 Ethics Statement

The video‐content analysis used publicly accessible material and involved neither interaction with uploaders nor the collection of private or identifiable data. The PPIE component comprised a one‐off consultation conducted through brief one‐to‐one discussions and was intended to inform the research process, not to collect individual‐level outcomes about caregivers. Participation was voluntary, and refusal did not affect clinical care. No identifiable personal or clinical information or individual quotations were retained.

### 2.2 Patient and Public Involvement and Engagement

We incorporated PPIE through a one‐off consultation comprising brief one‐to‐one discussions with family contributors. In line with the NIHR definition of consultation, we asked contributors for their views and used those views to inform specific research decisions [12]. The aim was to improve the public relevance of the study framing, lay terminology and parent‐facing interpretation, rather than to investigate caregiver perceptions, experiences or behaviour. Reporting followed GRIPP2 and other public‐involvement guidance [13, 14, 15].

On 10 August 2025, we approached 10 adult family contributors individually during pre‐anaesthesia assessment, using convenience sampling. The group included four mothers, three fathers, two grandparents and one primary caregiver. Brief one‐to‐one consultations sought advice on information sources relevant to the study and on understandable lay wording for concerns about paediatric general anaesthesia and child neurodevelopment. Contributors were approached after routine clinical communication had been completed or at another time that did not interfere with care. Participation was voluntary, and refusal did not affect the child's care. Recruitment did not depend on prior exposure to the videos later included in the study, and contributors did not review or score individual videos.

Contributors' advice supported the use of both professional terminology and a lay question‐based search phrase. It also helped the research team consider the parent‐facing relevance of omissions and false reassurance when interpreting the video findings. We used a PPIE consultation rather than a qualitative study because the purpose was to inform the research process, not to generate empirical findings about caregivers. The consultation was not designed to characterise caregiver experiences, estimate the prevalence of particular information sources or determine whether short videos affected understanding, anxiety or treatment decisions. We retained only anonymised, aggregate notes and did not collect individual quotations or undertake transcription, qualitative coding, thematic analysis or statistical analysis. Contributors did not participate in screening, scoring, misinformation classification or quantitative analysis. Further details are provided in Supporting Information S2: Table 1.

### 2.3 Search Strategy and Data Extraction

On 15 August 2025, we systematically searched Douyin (the Chinese version of TikTok) and Bilibili using two terms that represented professional language and lay concerns about anaesthesia‐related cognition: ‘儿童全身麻醉认知影响’ and ‘全麻会影响孩子智力吗’. To reduce personalisation bias, we used newly created accounts with no search or watch history, subscriptions, likes or other prior interactions. Search results were retained in each platform's default order, and advertisements were excluded before eligibility screening.

We collected the first 100 results from each platform because items near the top of search results are likely to be the most visible to users. Rank was defined as a video's ordinal position in the search results at the time of retrieval. For each eligible video, we recorded platform, uploader type, uploader category, duration, publication date and engagement metrics, including followers, likes, comments, collections (saves or bookmarks) and shares.

### 2.4 Eligibility Criteria

Videos were eligible if they contained substantive information about the potential cognitive or neurodevelopmental effects of paediatric general anaesthesia. We included content from professional and non‐professional uploaders when it addressed cognition, intelligence, memory, learning ability, brain development or related outcomes.

**Figure 1:**
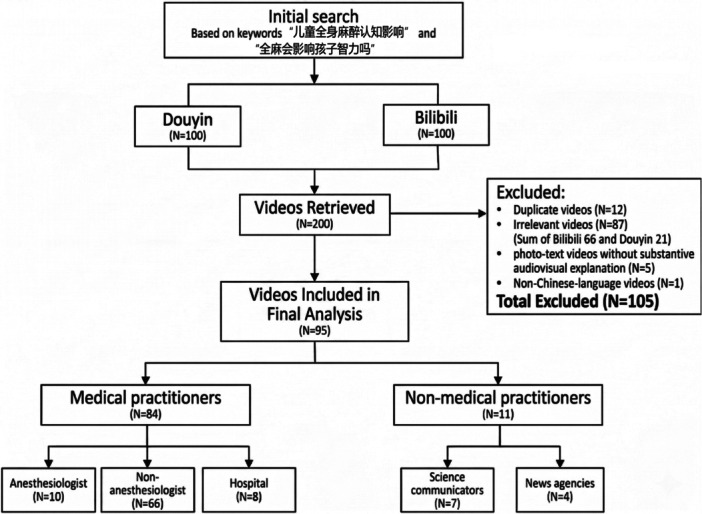
Video search strategy, eligibility screening and classification of included videos.

Videos were excluded if they were duplicates, advertisements, unrelated to the study topic, static image‐text posts without substantive audiovisual explanation, non‐Chinese‐language content, or inaccessible or incomplete recordings that could not be assessed adequately. The screening process is summarised in Figure 1.

### 2.5 Video Assessment

Two anaesthesiologists, Chong Xu and Yuchang Fang, independently assessed each video using the Global Quality Score (GQS), Medical Quality Video Evaluation Tool (MQ‐VET), JAMA benchmark criteria, modified DISCERN (mDISCERN) and the Patient Education Materials Assessment Tool for Audiovisual Materials (PEMAT‐A/V) [16, 17, 18, 19]. Operational definitions and scoring criteria are provided in Supporting Information S1: Appendix A. We combined these tools because no single instrument captures all relevant features of short‐form medical videos. A video may be clear and engaging yet poorly sourced, unbalanced or insufficiently explicit about uncertainty.

All evaluators had more than 10 years of clinical experience in anaesthesiology. Before formal scoring, the two primary evaluators completed standardised training and calibration. Inter‐rater agreement was assessed with Cohen's kappa and interpreted using the Landis and Koch criteria [20, 21]. For MQ‐VET and PEMAT understandability, which have broader ordinal score ranges, we used quadratically weighted kappa to reduce the influence of minor scoring differences. Disagreements were resolved by a third anaesthesiologist, Lei Yao. Misinformation was classified into three predefined groups: exaggeration and absolute claims, out‐of‐context interpretation and overgeneralisation, and false safety assurances.

### 2.6 Statistical Analysis

Statistical analyses were performed with IBM SPSS Statistics version 27.0 and Python 3.13; figures were prepared with GraphPad Prism version 9.0 and Matplotlib. Normality was assessed with the Shapiro–Wilk test. Non‐normally distributed continuous variables are presented as medians and interquartile ranges, and categorical variables as frequencies and percentages. Spearman's rank correlation was used for associations between non‐normally distributed continuous variables. Categorical variables were compared with the chi‐square test or Fisher's exact test, two independent groups with the Mann–Whitney *U* test and three or more groups with the Kruskal–Wallis test followed by Dunn's test. For stratified misinformation outcomes, we used Fisher's exact test for two‐group comparisons and the Fisher–Freeman–Halton exact test for uploader categories because several expected cell counts were small. The Benjamini–Hochberg method controlled the false discovery rate for multiple comparisons, including Dunn's tests, correlations and stratified misinformation analyses. All tests were two‐sided, with *α* = 0.05.

Effect sizes were calculated for group comparisons. For two‐group Mann–Whitney *U* tests, *r* was calculated as |*Z*| divided by the square root of the total sample size. For Kruskal–Wallis tests, epsilon‐squared was calculated as *ε* ^2^ = *H*/(*N* − 1), where *H* is the Kruskal–Wallis statistic and *N* the total analytic sample. Epsilon‐squared values were interpreted descriptively as negligible (< 0.01), small (0.01–0.059), moderate (0.060–0.139) or large (≥ 0.140). For Dunn's post hoc comparisons, pairwise *r* was calculated as |*Z*|/√*N* using the full analytic sample (*N* = 95). Values of *r* were interpreted as small, medium and large at approximate thresholds of 0.10, 0.30 and 0.50, respectively. Cramer's *V* was calculated for categorical misinformation comparisons and interpreted descriptively, particularly where sparse cells limited subgroup inference. Exact Spearman's rho coefficients, raw *p* values and Benjamini–Hochberg‐adjusted *p* values are reported in the supporting material.

We performed three sensitivity analyses to assess the effect of imbalanced subgroup sizes and highly skewed engagement metrics. First, because Douyin contributed more videos than Bilibili, we conducted platform‐balanced resampling. All 21 Bilibili videos were retained, and 21 Douyin videos were sampled without replacement in each of 1000 iterations. Platform comparisons were repeated with the Mann–Whitney *U* test, and the direction of effect, *p* value and *r* were recorded for each iteration.

Second, because several uploader categories were small, we repeated uploader analyses after combining categories into broader groups. Anaesthesiologist and hospital videos were grouped as anaesthesia‐specialist/institutional sources and compared with all other videos. In a further exploratory analysis, anaesthesiologist, hospital and news‐agency videos were grouped as specialist/institutional sources and compared with non‐specialist creators. Third, followers, likes, comments, collections and shares were transformed as log10(*x* + 1), and correlation analyses were repeated. These analyses assessed the robustness of the primary findings and did not replace the primary analyses.

## 3 Results

### 3.1 Overall Characteristics

Inter‐rater agreement was excellent for all assessment instruments. Cohen's kappa was 0.89 for mDISCERN, 0.88 for GQS, 0.89 for JAMA and 0.89 for PEMAT actionability. Quadratically weighted kappa was 0.96 for both MQ‐VET and PEMAT understandability.

Ninety‐five videos met the eligibility criteria: 74 from Douyin and 21 from Bilibili (Table 1). Most were uploaded by clinical or hospital sources (84/95, 88.4%), although non‐anaesthesiologists formed the largest individual uploader category (66/95, 69.5%). Anaesthesiologists uploaded 10 videos, hospitals 8, science communicators 7 and news agencies 4. Median duration was 58.0 seconds, and videos had been published a median of 549.0 days earlier. Engagement varied widely, with medians of 153 likes, 17 comments, 28 collections and 47 shares. Overall, the sample was dominated by short videos and non‐anaesthesiologist uploaders.

**Table 1:** Basic characteristics of included videos.

Variables	Total (*n* = 95)
Platform, *n* (%)	
Bilibili	21 (22.11)
Douyin	74 (77.89)
Uploader type, *n* (%)	
Clinical or hospital sources	84 (88.42)
Non‐medical sources	11 (11.58)
Uploader category, *n* (%)	
Non‐anaesthesiologist	66 (69.47)
Anaesthesiologist	10 (10.53)
Hospital official accounts	8 (8.42)
News agencies	4 (4.21)
Science communicators	7 (7.37)

*Note:* Values are *n* (%).

### 3.2 Comparisons by Platform

Douyin videos attracted substantially more engagement than Bilibili videos. Median follower, like, comment, collection and share counts were higher on Douyin, whereas Bilibili videos were longer (Table 2). Median likes were 182.5 on Douyin and 16.0 on Bilibili; median shares were 79.0 and 2.0, respectively. None of the quality measures differed significantly between platforms. Median mDISCERN, MQ‐VET, GQS, JAMA and PEMAT‐A/V scores were similar, indicating that greater reach was not accompanied by higher quality. Effect sizes for significant platform differences were small to medium for followers, likes, comments, collections and duration (*r* = 0.26–0.39), and medium for shares (*r* = 0.48). Effect sizes for differences in quality scores were negligible to small (*r* ≤ 0.11).

**Table 2:** Platform comparisons of video characteristics, engagement metrics and quality‐related scores.

Variable	Total (*n* = 95)	Bilibili (*n* = 21)	Douyin (*n* = 74)	*Z*	*p*	Effect size *r*
Followers	44,595.00 (10,887.50, 194,532.00)	9094.00 (217.00, 132,000.00)	60,993.00 (18,231.75, 196,101.00)	−2.52	0.012	0.259
Likes	153.00 (50.00, 492.50)	16.00 (3.00, 205.00)	182.50 (62.25, 575.00)	−3.49	< 0.001	0.358
Comments	17.00 (3.00, 80.50)	1.00 (0.00, 19.00)	26.50 (5.00, 91.50)	−3.13	0.002	0.321
Collections	28.00 (5.50, 86.00)	4.00 (2.00, 11.00)	33.50 (14.25, 99.25)	−3.79	< 0.001	0.389
Shares	47.00 (10.50, 186.50)	2.00 (1.00, 10.00)	79.00 (19.00, 211.00)	−4.69	< 0.001	0.481
Days since published	549.00 (317.50, 1066.50)	715.00 (467.00, 1420.00)	527.50 (311.50, 906.25)	−1.35	0.179	0.139
Duration	58.00 (48.50, 82.50)	83.00 (60.00, 149.00)	56.00 (44.50, 75.75)	−3.23	0.001	0.331
mDISCERN	2.00 (2.00, 3.00)	2.00 (2.00, 4.00)	2.00 (2.00, 3.00)	−0.72	0.473	0.074
MQ‐VET	40.00 (34.50, 47.00)	41.00 (32.00, 53.00)	40.00 (35.00, 46.75)	−0.04	0.964	0.004
PEMAT understandability	75.00 (67.00, 80.00)	70.00 (64.00, 82.00)	75.00 (67.00, 80.00)	−0.08	0.939	0.008
PEMAT actionability	67.00 (67.00, 75.00)	67.00 (50.00, 67.00)	67.00 (67.00, 75.00)	−1.09	0.277	0.112
GQS	3.00 (3.00, 4.00)	3.00 (2.00, 4.00)	3.00 (3.00, 4.00)	−0.34	0.731	0.035
JAMA	1.00 (0.00, 1.00)	1.00 (0.00, 1.00)	1.00 (0.00, 1.00)	−0.32	0.748	0.033

*Note:* Values are median (Q1, Q3). Mann–Whitney *U* tests were used; *r* = |*Z*|/sqrt(*N*), *N* = 95.

### 3.3 Comparisons by Uploader Group and Category

Videos from clinical or hospital sources had significantly higher follower counts, likes, comments, collections and shares than videos from non‐medical sources. Videos from non‐medical sources were significantly longer. The two broad uploader groups did not differ significantly in GQS, mDISCERN, MQ‐VET, JAMA, PEMAT understandability or PEMAT actionability (Table 3). Effect sizes for engagement and duration were small to medium (*r* = 0.20–0.32), whereas those for quality scores were negligible to small (*r* ≤ 0.10).

**Table 3:** Comparisons by broad uploader group.

Variable	Total (*n* = 95)	Clinical or hospital sources (*n* = 84)	Non‐medical sources (*n* = 11)	*Z*	*p*	Effect size *r*
Followers	44,595.00 (10,887.50, 194,532.00)	57,803.50 (14,371.50, 192,963.00)	9094.00 (144.00, 160,000.00)	−1.99	0.046	0.204
Likes	153.00 (50.00, 492.50)	178.00 (57.00, 516.50)	4.00 (2.50, 48.50)	−3.11	0.002	0.319
Comments	17.00 (3.00, 80.50)	23.50 (4.75, 90.50)	1.00 (0.00, 5.50)	−2.89	0.004	0.297
Collections	28.00 (5.50, 86.00)	31.00 (11.00, 92.50)	3.00 (2.00, 11.00)	−2.72	0.006	0.279
Shares	47.00 (10.50, 186.50)	51.50 (15.50, 190.25)	2.00 (1.50, 13.50)	−2.66	0.008	0.273
Days since published	549.00 (317.50, 1066.50)	527.50 (318.25, 902.75)	1238.00 (369.00, 1613.00)	−1.47	0.141	0.151
Duration	58.00 (48.50, 82.50)	57.00 (46.75, 76.00)	122.00 (76.00, 156.50)	−2.93	0.003	0.301
mDISCERN	2.00 (2.00, 3.00)	2.00 (2.00, 3.00)	3.00 (2.00, 4.50)	−1.01	0.313	0.104
MQ‐VET	40.00 (34.50, 47.00)	40.00 (35.00, 47.00)	41.00 (32.00, 56.00)	−0.01	0.991	0.001
PEMAT understandability	75.00 (67.00, 80.00)	75.00 (67.00, 80.00)	77.00 (67.00, 80.00)	−0.26	0.797	0.027
PEMAT actionability	67.00 (67.00, 75.00)	67.00 (67.00, 67.00)	67.00 (58.50, 75.00)	−0.60	0.550	0.062
GQS	3.00 (3.00, 4.00)	3.00 (3.00, 4.00)	3.00 (2.50, 4.00)	−0.35	0.730	0.036
JAMA	1.00 (0.00, 1.00)	1.00 (0.00, 1.00)	1.00 (0.00, 1.00)	−0.26	0.793	0.027

*Note:* Values are median (Q1, Q3). Mann–Whitney *U* tests were used; *r* = |*Z*|/sqrt(*N*), *N* = 95.

**Figure 2:**
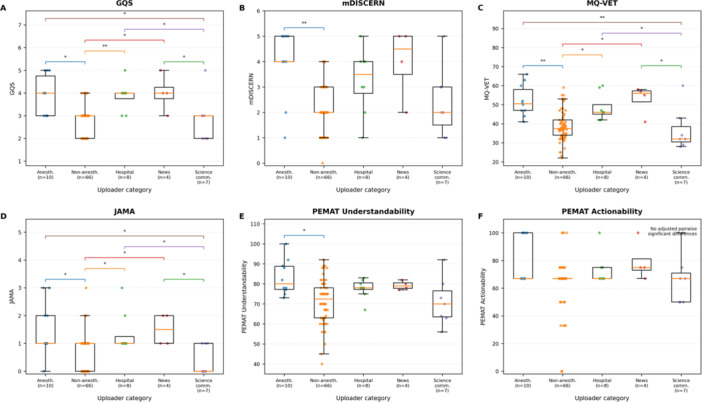
Quality‐related scores across uploader categories. (A) Global Quality Score (GQS); (B) modified DISCERN (mDISCERN); (C) Medical Quality Video Evaluation Tool (MQ‐VET); (D) JAMA benchmark criteria; (E) Patient Education Materials Assessment Tool for Audiovisual Materials (PEMAT‐A/V) understandability; and (F) PEMAT‐A/V actionability. Boxplots show medians and interquartile ranges; jittered points represent individual videos. Pairwise comparisons were performed using Dunn post hoc tests after Kruskal–Wallis tests, and *p* values were adjusted using the Benjamini–Hochberg method. Only statistically significant adjusted comparisons are displayed (*p < 0.05, **p < 0.01 and ***p < 0.001). Full pairwise results are provided in Supporting Information S2: Table 2. Category‐level comparisons are exploratory because several subgroups were small.

Further stratification by uploader category showed significant overall differences in likes, comments, collections, duration, mDISCERN, MQ‐VET, GQS, JAMA, PEMAT understandability and PEMAT actionability (Table 4 and Figure 2). Epsilon‐squared values were largest for quality measures, particularly MQ‐VET (*ε* ^2^ = 0.305), GQS (*ε* ^2^ = 0.256), JAMA (*ε* ^2^ = 0.219) and mDISCERN (*ε* ^2^ = 0.218). Effect sizes for engagement and descriptive variables were more variable: likes (*ε* ^2^ = 0.139), comments (*ε* ^2^ = 0.168), collections (*ε* ^2^ = 0.115) and duration (*ε* ^2^ = 0.199). Uploader category was therefore more strongly associated with quality and reliability than with most engagement measures. Full Dunn post hoc results, including *Z* values, raw and adjusted *p* values and pairwise effect sizes, are reported in Supporting Information S2: Table 2.

**Table 4:** Comparisons by uploader category.

Variable	Total (*n* = 95)	Anaesth. (*n* = 10)	Hospital (*n* = 8)	News agencies (*n* = 4)	Non‐anaesth. (*n* = 66)	Science communicators (*n* = 7)	*H*	*p*	*ε* ^2^	Magnitude
Followers	44,595.00 (10,887.50, 194,532.00)	36,417.00 (16,745.25, 245,715.00)	44,489.00 (23,588.00, 144,489.25)	158,047.00 (6822.75, 1,276,922.75)	64,053.50 (14,706.00, 196,101.00)	1952.00 (144.00, 11,272.50)	6.54	0.162	0.070	Moderate
Likes	153.00 (50.00, 492.50)	627.50 (106.00, 1615.00)	84.50 (55.00, 180.25)	15.50 (2.50, 1049.50)	182.50 (56.50, 448.50)	4.00 (2.50, 38.00)	13.11	0.011	0.139	Moderate
Comments	17.00 (3.00, 80.50)	148.00 (19.75, 351.50)	2.00 (1.50, 9.00)	0.50 (0.00, 69.75)	23.50 (5.00, 69.50)	1.00 (0.00, 5.50)	15.83	0.003	0.168	Large
Collections	28.00 (5.50, 86.00)	79.50 (10.25, 224.75)	19.50 (4.75, 29.75)	3.00 (3.00, 437.00)	32.50 (12.25, 88.50)	2.00 (1.00, 11.00)	10.80	0.029	0.115	Moderate
Shares	47.00 (10.50, 186.50)	78.50 (11.50, 431.25)	49.00 (11.00, 89.50)	2.00 (1.75, 276.25)	64.00 (16.00, 188.25)	2.00 (1.50, 13.50)	8.02	0.091	0.085	Moderate
Days since published	549.00 (317.50, 1066.50)	831.50 (571.50, 1145.25)	635.50 (399.75, 1025.25)	1026.00 (413.50, 1597.25)	499.50 (309.25, 863.00)	1238.00 (487.50, 1838.50)	5.32	0.256	0.057	Small
Duration	58.00 (48.50, 82.50)	94.50 (69.25, 177.50)	66.00 (46.75, 76.75)	156.50 (130.75, 272.75)	56.00 (44.50, 65.00)	86.00 (64.00, 127.50)	18.71	< 0.001	0.199	Large
mDISCERN	2.00 (2.00, 3.00)	4.00 (4.00, 5.00)	3.50 (2.75, 4.00)	4.50 (3.50, 5.00)	2.00 (2.00, 3.00)	2.00 (1.50, 3.00)	20.51	< 0.001	0.218	Large
MQ‐VET	40.00 (34.50, 47.00)	50.50 (47.00, 58.00)	46.00 (45.00, 50.00)	56.00 (51.50, 57.25)	37.50 (34.00, 42.00)	32.00 (30.50, 38.50)	28.71	< 0.001	0.305	Large
PEMAT understandability	75.00 (67.00, 80.00)	80.00 (77.25, 88.75)	78.00 (77.25, 80.50)	79.00 (77.75, 80.50)	72.50 (63.00, 78.00)	70.00 (63.50, 76.50)	14.37	0.006	0.153	Large
PEMAT actionability	67.00 (67.00, 75.00)	67.00 (67.00, 100.00)	67.00 (67.00, 75.00)	75.00 (73.00, 81.25)	67.00 (67.00, 67.00)	67.00 (50.00, 71.00)	10.37	0.035	0.110	Moderate
GQS	3.00 (3.00, 4.00)	4.00 (3.00, 4.75)	4.00 (3.75, 4.00)	4.00 (3.75, 4.25)	3.00 (2.00, 3.00)	3.00 (2.00, 3.00)	24.03	< 0.001	0.256	Large
JAMA	1.00 (0.00, 1.00)	1.00 (1.00, 2.00)	1.00 (1.00, 1.25)	1.50 (1.00, 2.00)	1.00 (0.00, 1.00)	0.00 (0.00, 1.00)	20.58	< 0.001	0.219	Large

*Note:* Values are median (Q1, Q3). Kruskal–Wallis *H* tests were used. Epsilon‐squared = *H*/(*N* − 1), *N* = 95. Full adjusted Dunn comparisons are in Supporting Information S2: Table 2.

Abbreviations: Anaesth., anaesthesiologist; non‐anaesth., non‐anaesthesiologist.

After Benjamini–Hochberg adjustment, anaesthesiologist videos scored higher than non‐anaesthesiologist videos on mDISCERN, MQ‐VET, GQS, JAMA and PEMAT understandability. In selected comparisons, hospital and news‐agency videos also scored higher than non‐anaesthesiologist and science‐communicator videos on MQ‐VET, GQS and JAMA. No pairwise difference in PEMAT actionability remained significant after adjustment. Significant comparisons are shown in Figure 2, with full Dunn statistics and effect sizes in Supporting Information S2: Table 2. Because the anaesthesiologist, hospital, news‐agency and science‐communicator groups were small, category‐level comparisons should be considered exploratory.

### 3.4 Misinformation Characteristics

Three misinformation patterns were identified (Supporting Information S2: Table 3). At least one predefined type of misinformation appeared in 51 videos (53.7%). False safety assurances were most common, occurring in 43 videos (45.3%), mainly because potential risks were not presented in a balanced way or key modifying variables were omitted. Out‐of‐context interpretation and overgeneralisation occurred in 5 videos (5.3%), including misinterpretation of FDA warnings, direct extrapolation from animal studies to children and generalisation of repeated or prolonged exposure to all anaesthetic scenarios. Exaggeration and absolute claims occurred in 3 videos (3.2%), including statements that a single brief exposure inevitably causes brain damage or permanently impairs intelligence or memory.

No platform difference remained significant after false‐discovery‐rate correction. Any misinformation occurred in 47.6% of Bilibili videos and 55.4% of Douyin videos; false safety assurances occurred in 38.1% and 47.3%, respectively (Supporting Information S2: Table 4). Broad uploader group was also not significantly associated with misinformation. The comparison across detailed uploader categories for any misinformation had a moderate Cramer's *V* (0.322), but was no longer significant after correction (raw *p* = 0.030; adjusted *p* = 0.355). These subgroup patterns were interpreted descriptively.

### 3.5 Correlation Analysis

**Figure 3:**
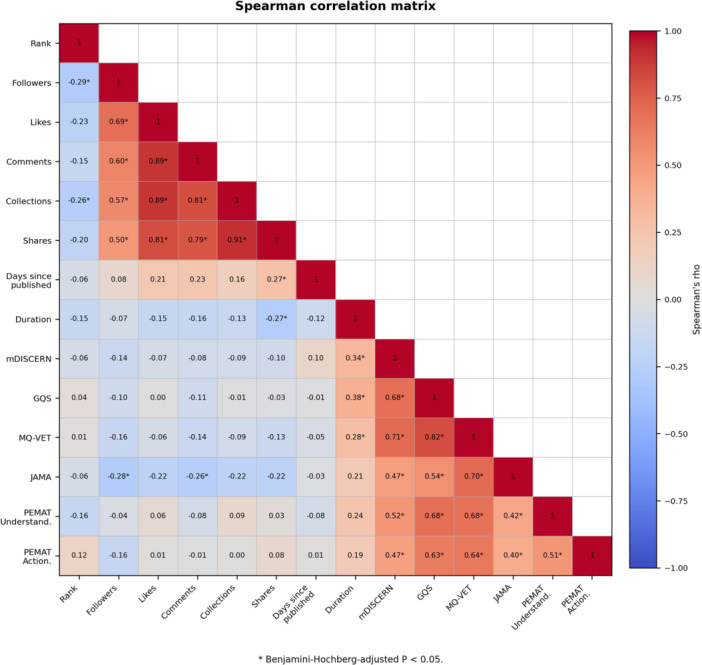
Spearman correlation matrix of video characteristics, engagement metrics and quality‐related scores. Values are Spearman's rho; asterisks indicate Benjamini–Hochberg‐adjusted *p* < 0.05. Exact coefficients, raw *p* values and adjusted *p* values are provided in Supporting Information S2: Table 5.

Spearman correlation analysis examined 91 unique pairwise associations among video characteristics, engagement metrics and quality scores (Figure 3 and Supporting Information S2: Table 5). After Benjamini–Hochberg correction, 34 associations remained significant. Engagement measures were strongly intercorrelated. Likes correlated strongly with comments (rho = 0.891, adjusted *p* < 0.001), collections (rho = 0.892, adjusted *p* < 0.001) and shares (rho = 0.814, adjusted *p* < 0.001); collections also correlated strongly with shares (rho = 0.908, adjusted *p* < 0.001). Search rank had weak negative correlations with followers (rho = −0.287, adjusted *p* = 0.016) and collections (rho = −0.258, adjusted *p* = 0.031), indicating that videos appearing nearer the top of the results tended to come from accounts with more followers and to have more collections. Associations with likes, comments and shares did not remain significant after correction.

Engagement was not a reliable indicator of informational quality. No positive association between engagement and a quality score remained significant after correction. Only two weak inverse associations were significant: followers with JAMA score (rho = −0.277, adjusted *p* = 0.020) and comments with JAMA score (rho = −0.261, adjusted *p* = 0.029). Video duration was positively associated with mDISCERN (rho = 0.335, adjusted *p* = 0.003), GQS (rho = 0.381, adjusted *p* < 0.001) and MQ‐VET (rho = 0.285, adjusted *p* = 0.016), but not with JAMA, PEMAT understandability or PEMAT actionability after correction. Scores on the quality instruments were moderately to strongly correlated, including GQS with MQ‐VET (rho = 0.816, adjusted *p* < 0.001), MQ‐VET with JAMA (rho = 0.704, adjusted *p* < 0.001) and mDISCERN with MQ‐VET (rho = 0.706, adjusted *p* < 0.001).

### 3.6 Sensitivity Analyses

Platform‐balanced resampling supported the primary findings (Supporting Information S2: Table 6). Higher engagement on Douyin persisted in all 1000 iterations for likes, comments, collections and shares, and the corresponding differences were significant in 94.5%–100% of iterations. Bilibili videos were longer in every iteration, with a significant difference in 86.9%. No consistent platform difference emerged for any quality score.

Collapsed‐category analyses generally showed higher mDISCERN, MQ‐VET, GQS, JAMA and PEMAT understandability scores for anaesthesia‐specialist/institutional sources, with mostly medium effect sizes. Log10(*x* + 1) transformation of engagement measures did not materially alter their strong intercorrelations or weak associations with quality (Supporting Information S2: Tables 7 and 8).

## 4 Discussion

Douyin videos attracted more engagement than Bilibili videos, but quality did not differ by platform. Uploader category was more informative: videos from anaesthesiologists and hospitals generally performed better than those from non‐anaesthesiologists in several domains, and news‐agency videos also performed well in some comparisons. Misinformation was common and most often took the form of false reassurance rather than overt alarmism. Popularity was a poor guide to quality, whereas longer videos tended to score modestly better. Overall, reach and informational quality were only weakly related.

Caregivers may encounter short videos before meeting an anaesthesiologist, but this study did not identify individual viewers or measure actual exposure to the included content. We therefore cannot determine how often, how strongly or in what direction these videos inform understanding, anxiety, trust or decisions. Nevertheless, the findings identify incomplete and binary claims that clinicians may need to clarify during preoperative consultation. Asking families whether they have encountered online information and which claims they remember may provide a useful starting point for individualised risk communication. The PPIE consultation informed the study framing and parent‐facing interpretation but did not provide evidence about caregiver exposure or behavioural outcomes.

After adjustment, uploader‐related differences were most consistent for MQ‐VET, GQS and JAMA. Anaesthesiologist, hospital and news‐agency videos scored higher than non‐anaesthesiologist or science‐communicator videos in selected comparisons. Differences in mDISCERN and PEMAT were less consistent, and no pairwise difference in PEMAT actionability remained significant. These findings do not support a definitive ranking of uploader groups, but they suggest that specialty expertise and institutional production may improve structure, attribution and overall educational quality. Lower scores among non‐anaesthesiologist and science‐communicator videos do not imply that these groups should avoid public education. Rather, anaesthesia‐specific expertise, visible credentials, source citation and acknowledgement of uncertainty appear especially important when communicating neurodevelopmental risk.

These uploader differences should be viewed in the context of perioperative care. Surgical need and timing may be discussed before the anaesthesia consultation, so anaesthesiologists often address expectations already shaped by surgeons, relatives and online material. Research on anaesthesiologist‐patient communication and informed consent indicates that patients value clear, individualised explanations and opportunities to ask questions [22, 23]. Parent‐facing videos should identify the speaker's credentials, explain the anaesthesiologist's perioperative role, distinguish anaesthetic risk from surgical necessity and encourage personalised consultation.

The misinformation findings are directly relevant to counselling. The main problem was not dramatic alarmism but oversimplified reassurance. Many videos omitted important qualifiers, including age at exposure, single versus repeated exposure, prolonged duration and the distinction between animal evidence and human clinical data. Such omissions can reduce nuanced evidence to binary messages of complete safety or inevitable harm. Current evidence supports a more qualified explanation: single, brief exposures have not consistently been associated with measurable global cognitive impairment, whereas repeated or prolonged exposures in very young children require individualised discussion of age, exposure duration, surgical necessity and the risks of delaying an indicated procedure [1, 2, 3, 4, 5, 6].

False reassurance warrants particular attention because it may sound more acceptable than fear‐based misinformation. A claim that general anaesthesia is completely harmless may be comforting, but it may weaken the informational basis of consent by omitting uncertainty and exposure‐related modifiers. Conversely, a claim that any exposure inevitably harms intelligence could increase anxiety or contribute to unnecessary delay. Both messages restrict shared understanding. Clinicians should replace neither fear nor uncertainty with blanket reassurance, but explain what is known, what remains uncertain, which factors modify risk and why the procedure is being considered at that time.

Stratified analyses suggested that oversimplified reassurance was not confined to one platform or broad uploader group. Interventions directed at a single platform may therefore miss a wider problem: nuanced evidence is often compressed into binary messages. Variation across detailed uploader categories should not be treated as a stable ranking because subgroup sizes were small and no difference remained significant after correction.

Shared decision‐making helps explain the clinical relevance of these findings. It requires more than a reassuring or cautionary conclusion; caregivers and clinicians need a shared understanding of the evidence, available options, uncertainties, trade‐offs and family preferences [24]. In this study, false safety assurance was the dominant misinformation pattern, and videos often omitted contextual qualifiers such as age, single versus repeated exposure, prolonged duration and surgical necessity. These omissions can reduce the discussion to whether anaesthesia is simply ‘safe’ or ‘harmful’, rather than supporting an individualised assessment of what is known, what remains uncertain, why the procedure is needed and how its benefits and risks should be weighed. Clinicians can address this by identifying the specific online claim, acknowledging the concern behind it and restoring the context needed for a balanced discussion.

The Health Belief Model offers a second way to understand why these content patterns matter [25]. Alarmist claims that any anaesthetic exposure inevitably causes brain injury could heighten perceived susceptibility and severity, whereas categorical reassurance that anaesthesia is entirely harmless could reduce perceived susceptibility and attention to modifying factors. Videos that present a conclusion without explaining uncertainty, individual circumstances or when to seek professional advice may also provide weak or inappropriate cues to action. These are interpretive possibilities rather than demonstrated effects: the study did not measure actual exposure, perceived risk, anxiety or decision outcomes. The model nevertheless helps explain why exaggeration, omission and false reassurance are relevant to preoperative communication.

Visible popularity metrics raise a separate credibility issue. Users may rely on follower counts, likes, comments or shares as quick heuristics when they cannot readily assess the underlying evidence [26]. In this study, however, no positive association between engagement and quality remained significant after correction. Popularity should not be treated as a proxy for informational quality. During consultation, asking which claim a caregiver encountered may be more useful than assuming that widely circulated content is either credible or uniformly misleading.

The five assessment tools highlighted different aspects of video quality. GQS measured overall educational flow and usefulness; mDISCERN emphasised reliability, balance, sources and uncertainty; JAMA focused on authorship, attribution, disclosure and currency; MQ‐VET added educational content, production quality and viewer experience; and PEMAT‐A/V separated understandability from actionability. Together, the tools showed that a video could be easy to understand and persuasive while remaining poorly sourced, unbalanced or vague about uncertainty. Clarity should therefore not be mistaken for credibility. Using several tools helped distinguish communicative accessibility from evidential trustworthiness.

The implications span clinical practice, institutional communication and platform governance. Anaesthesiologists should ask about online information, distinguish a single brief exposure from repeated or prolonged exposure and discuss anaesthetic risk alongside surgical necessity. Hospital education teams should work with families and multidisciplinary clinicians to produce short videos that identify authorship, dates and sources. Platforms should strengthen credential verification, source labelling, conflict‐of‐interest disclosure and links to authoritative resources, particularly for absolute safety or harm claims. Where feasible, future materials should involve families beyond a one‐off consultation and move towards co‐production, in keeping with public‐involvement guidance [12, 13, 14, 15].

In practice, a brief consultation sequence may help. The anaesthesiologist can begin by asking whether the family has seen online information about anaesthesia and brain development, then identify the exact claim rather than trying to correct ‘the internet’ in general. The response can distinguish what is relatively reassuring from what remains uncertain: current human evidence is reassuring regarding global cognitive outcomes after a single brief exposure, whereas repeated or prolonged exposure in very young children requires individualised discussion. The clinician can then relate this information to the child's indication and timing of surgery, expected exposure, relevant comorbidity and the possible consequences of delay. Finally, families can be invited to restate the main message and raise remaining concerns. This approach is intended not to lengthen every consultation, but to identify misconceptions early and focus limited time on the information shaping the family's questions. It also avoids dismissing online information because of its source or countering fear with unqualified reassurance.

Parent‐facing videos should be developed as clinical education products rather than isolated social‐media posts. At minimum, they should identify a clinical author and institutional affiliation, provide a publication or review date and concise source attribution, disclose relevant interests, state uncertainty explicitly and prompt viewers to seek individual advice. Content review should involve anaesthesiologists, surgeons, nurses, communication specialists and family contributors so that scientific accuracy, readability and practical usefulness are considered together. Hospitals can link these materials to preoperative booking messages, clinic webpages and consent resources, while ensuring that videos supplement rather than replace discussion. Platforms can support this work through credential verification, visible source labels and links to authoritative resources. Moderation should focus on demonstrably absolute or misleading claims rather than treating all disagreement or uncertainty as misinformation. Implementation also requires evaluation. Relevant outcomes include completion and retention rates, understanding of exposure‐related modifiers, confidence in asking questions, consultation efficiency and unintended effects such as increased anxiety. Without such evaluation, professionally produced content may appear credible yet still fail to support understanding or action.

Strengths of this study include the assessment of two major short‐video platforms within the same clinical topic, the use of newly created accounts to reduce personalisation bias, multiple complementary quality instruments, predefined misinformation categories and sensitivity analyses. The one‐off PPIE consultation also helped contextualise the parent‐facing relevance of the findings. These design choices allowed us to compare reach with objective measures of content quality and to identify uploader groups that tended to perform better in selected quality domains.

Several limitations should be considered. First, the findings reflect a single retrieval date in a dynamic algorithmic environment. Search rankings, engagement metrics and available content may change, and newly created accounts cannot reproduce the personalised feeds seen by caregivers with established viewing histories. Platform patterns may therefore not be stable over time or reproducible under different account conditions. Second, the analysis was limited to Chinese‐language content on Douyin and Bilibili. Differences in platform design, governance, cultural risk communication and online health‐information use limit generalisability beyond this media environment. Third, viewer identities and motivations were unknown. Engagement counts cannot establish whether viewers were caregivers of children undergoing surgery, whether they watched complete videos or whether the content affected anxiety, trust, consent or treatment decisions. Fourth, the sample was modest and unevenly distributed across platforms and uploader categories. Several uploader subgroups contained few videos, which reduced precision and statistical power. Exact tests, false‐discovery‐rate correction, effect‐size reporting and sensitivity analyses reduced but did not eliminate this uncertainty; detailed uploader‐category findings remain exploratory. Fifth, some subjectivity is unavoidable in content assessment despite high inter‐rater agreement. Finally, the PPIE component comprised brief one‐to‐one consultations with a convenience sample of 10 contributors from a single pre‐anaesthesia assessment setting. It provided limited advisory input and was not intended to generate representative qualitative evidence, estimate online information exposure or establish effects on caregiver behaviour.

Future research should distinguish the availability of online content from actual caregiver exposure and subsequent outcomes. Longitudinal platform studies could repeat identical searches at prespecified intervals and across different account histories, geographic settings, languages and platforms. Caregiver studies should record which materials were viewed, when they were viewed and which claims were remembered before assessing knowledge, anxiety, trust, decisional conflict and treatment preferences. Randomised or pragmatic trials could compare usual information with co‐produced, anaesthesiologist‐led videos delivered before consultation. Relevant outcomes include understanding of single versus repeated or prolonged exposure, recognition of uncertainty, the quality of questions raised, consultation duration, decisional conflict, treatment delay and satisfaction. Formal qualitative studies could examine how caregivers reconcile conflicting messages from clinicians, relatives and online sources.

## 5 Conclusion

Public‐facing videos about paediatric general anaesthesia and neurodevelopment were readily accessible but uneven in reliability and transparency. Popularity did not indicate quality, and false safety assurance was the most common misinformation pattern. Specialist or institutional involvement may improve several quality domains, although detailed uploader‐category findings remain exploratory. Parent‐centred counselling should invite discussion of online information, correct binary claims, explain exposure‐related modifiers and direct families to trustworthy clinician‐produced resources.

## Author Contributions

**Chong Xu:** formal analysis, investigation, data curation. **Yuchang Fang:** formal analysis, investigation, data curation. **Lei Yao:** project administration, resources, writing – review and editing, funding acquisition, supervision. **Jie Song:** supervision, writing – review and editing, project administration.

## Ethics Statement

The video‐content analysis used publicly accessible material and involved neither interaction with uploaders nor the collection of private or identifiable data. The PPIE component was an advisory consultation that informed study framing and parent‐facing interpretation; no identifiable personal or clinical information, individual quotations or individual‐level research outcomes were retained.

## Conflicts of Interest

The authors declare no conflicts of interest.

## Patient or Public Contribution

Ten family contributors took part in brief one‐to‐one PPIE consultations during pre‐anaesthesia assessment. Their advice supported the use of lay search language and the parent‐facing interpretation of omissions and false reassurance. Contributors did not review or score videos, and their views were not treated as individual‐level research data. Their contribution is acknowledged in the Acknowledgements and reported using GRIPP2 in Supporting Information S2: Table 1.

## Supporting information


Supporting File 1

Supporting File 2

## Data Availability

The data supporting the video analysis are available from the corresponding author upon reasonable request. The PPIE consultation did not generate an individual‐level research dataset; only anonymised, aggregate notes were retained.
