# Supplementary material for: Public‐Facing Short Videos on Paediatric General Anaesthesia and Child Neurodevelopment: Quality, Misinformation and Implications for Parent‐Centred Risk Communication
Source: Health Expect. 2026 Aug 17;29(4):e70826. doi: 10.1111/hex.70826 (PMC13478835; doi:10.1111/hex.70826)
Supplement: Supplementary file 1 [file HEX-29-e70826-s001.docx]

Supplementary Appendix A

Public-Facing Short Videos on Paediatric General Anaesthesia and Child Neurodevelopment: Quality, Misinformation, and Implications for Parent-Centred Risk Communication

This appendix provides the operational definitions of uploader categories and the assessment criteria used to evaluate video quality, reliability, transparency, understandability, and actionability.

Appendix Table A1. Categories and definitions of uploaders

| Classification level | Category | Definition |
| --- | --- | --- |
| Uploader type | Clinical or hospital sources | Anaesthesiologists, non-anaesthesiologist clinicians, and official hospital or department accounts. |
| Uploader type | Non-medical sources | Science communicators and news-agency accounts without direct clinical responsibility for the topic. |
| Uploader category | Anaesthesiologist | Licensed physicians specialized in anaesthesiology or perioperative medicine who provide topic-specific professional interpretation of paediatric general anaesthesia. |
| Uploader category | Non-anaesthesiologist | Clinical or hospital sources from specialties other than anaesthesiology, such as paediatricians, surgeons, nurses, or other clinicians, who discuss the potential cognitive effects of paediatric general anaesthesia. |
| Uploader category | Hospital | Official accounts of hospitals, hospital departments, or affiliated clinical institutions that disseminate health information in an organizational capacity. |
| Uploader category | News agencies | Official media or news organization accounts that publish health-related information for public communication and reporting. |
| Uploader category | Science communicators | Individual or team-operated accounts focused on science or health popularization, usually emphasizing public explanation rather than direct clinical care. |

Note. Definitions were tailored to the uploader taxonomy used in the present study.

Appendix Table A2. Global Quality Score (GQS) criteria

| Score | Description |
| --- | --- |
| 1 | Poor quality, poor flow, most information missing, and not at all useful for patients. |
| 2 | Generally poor quality and poor flow, with some information listed but many important topics missing; of very limited use to patients. |
| 3 | Moderate quality with suboptimal flow; some important information is adequately discussed but other important topics are poorly discussed; somewhat useful for patients. |
| 4 | Good quality and generally good flow; most relevant information is presented, although some topics are not covered; useful for patients. |
| 5 | Excellent quality and excellent flow; very useful for patients. |

Note. GQS is a 5-point global measure of overall educational quality and flow. Higher scores indicate better educational usefulness.

Appendix Table A3. Modified DISCERN (mDISCERN) criteria

| Item | Criterion |
| --- | --- |
| 1 | Is the video clear, concise, and understandable? |
| 2 | Are valid sources cited? |
| 3 | Is the content presented in a balanced and unbiased manner? |
| 4 | Are additional sources of information listed for patient reference? |
| 5 | Are areas of uncertainty mentioned? |

Note. Each item is scored 1 for “yes” and 0 for “no”. Total score range: 0–5; higher scores indicate greater reliability.

Appendix Table A4. Medical Quality Video Evaluation Tool (MQ-VET) criteria

| Domain | Item | Criterion |
| --- | --- | --- |
| Part 1: Reliability and transparency | 1 | Dates of updates, if any, are clearly stated. |
| Part 1: Reliability and transparency | 2 | The recording date of the video and the date on which the information was accessed are mentioned. |
| Part 1: Reliability and transparency | 3 | The resources and references used are clearly stated. |
| Part 1: Reliability and transparency | 4 | Concerns about advertising and potential conflicts of interest have been addressed. |
| Part 1: Reliability and transparency | 5 | Sufficient information is provided about the identity of the presenter in the video. |
| Part 2: Educational content | 6 | The materials used in the video facilitate learning. |
| Part 2: Educational content | 7 | The video covers the basic concepts of the subject. |
| Part 2: Educational content | 8 | Visual resources are used sufficiently to explain the medical topic. |
| Part 2: Educational content | 9 | Medical terms are well explained. |
| Part 3: Production quality | 10 | The sound quality of the video is sufficient. |
| Part 3: Production quality | 11 | The image quality of the video is sufficient. |
| Part 3: Production quality | 12 | The information in the video is clear and understandable. |
| Part 4: Viewer experience | 13 | The video generally meets the evaluator's expectations. |
| Part 4: Viewer experience | 14 | Information about the video content is provided at the beginning. |
| Part 4: Viewer experience | 15 | The video provides new knowledge and skills. |

Note. Each item is rated on a 5-point Likert scale (1 = strongly disagree to 5 = strongly agree). Total score range: 15–75; higher scores indicate better medical video quality.

Appendix Table A5. JAMA benchmark criteria

| Criterion | Description |
| --- | --- |
| Authorship | Authors and contributors, together with their affiliations and relevant credentials, should be identified. |
| Attribution | References and sources for the content should be clearly listed, and relevant copyright information should be indicated. |
| Disclosure | Ownership, sponsorship, advertising, commercial support, funding, and potential conflicts of interest should be clearly disclosed. |
| Currency | The initial posting date and the dates of updates should be indicated. |

Note. Each criterion is scored as present (1) or absent (0). Total score range: 0–4; higher scores indicate greater transparency and reliability.

Appendix Table A6. PEMAT-A/V criteria for audiovisual materials

| Domain | Item | Criterion |
| --- | --- | --- |
| Understandability | 1 | The material makes its purpose completely evident. |
| Understandability | 2 | The material uses common, everyday language. |
| Understandability | 3 | Medical terms are used only to familiarize the audience with the terms; when used, medical terms are defined. |
| Understandability | 4 | The material uses the active voice. |
| Understandability | 5 | The material breaks or chunks information into short sections. |
| Understandability | 6 | The material's sections have informative headers. |
| Understandability | 7 | The material presents information in a logical sequence. |
| Understandability | 8 | The material provides a summary. |
| Understandability | 9 | The material uses visual cues to draw attention to key points. |
| Understandability | 10 | Text shown on the screen is easy to read. |
| Understandability | 11 | The material allows the user to hear the words clearly. |
| Understandability | 12 | The material uses illustrations and photographs that are clear and uncluttered. |
| Understandability | 13 | The material uses simple tables with short and clear row and column headings. |
| Actionability | 14 | The material clearly identifies at least one action the user can take. |
| Actionability | 15 | The material addresses the user directly when describing actions. |
| Actionability | 16 | The material breaks down any action into manageable, explicit steps. |
| Actionability | 17 | The material explains how to use charts, graphs, tables, or diagrams to take action. |

Note. For PEMAT-A/V, each applicable item is rated as Agree (1) or Disagree (0); some items may be marked Not Applicable. Separate percentage scores are calculated for understandability (13 items) and actionability (4 items). Higher percentages indicate better performance.
