# Supplementary material for: Public‐Facing Short Videos on Paediatric General Anaesthesia and Child Neurodevelopment: Quality, Misinformation and Implications for Parent‐Centred Risk Communication
Source: Health Expect. 2026 Aug 17;29(4):e70826. doi: 10.1111/hex.70826 (PMC13478835; doi:10.1111/hex.70826)
Supplement: Supplementary file 2 [file HEX-29-e70826-s002.docx]

Supplementary material

Supplementary Table S1. GRIPP2-informed reporting of patient and public involvement and engagement.

| **GRIPP2 reporting item** | **Reporting in the present study** |
| --- | --- |
| Aim of PPIE | The PPIE component was a one-off consultation comprising brief one-to-one discussions with family contributors. It sought advice on the public relevance of the research question, information sources relevant to study framing, understandable lay terminology and the parent-facing interpretation of the video findings. In line with the NIHR consultative approach, contributors’ views informed research decisions rather than generating empirical findings about caregiver perceptions, experiences or behaviour. |
| Stage of involvement | PPIE took place before the formal platform search and video assessment. The consultation informed study framing, lay terminology and the parent-facing interpretation of the findings. Family contributors did not participate in video screening, scoring, misinformation classification, statistical analysis or interpretation of individual platform or uploader-category comparisons. |
| Contributor characteristics | Ten adult family contributors of children scheduled for surgery requiring anaesthesia took part. They were involved in preoperative decision-making or information seeking for the child and included parents, grandparents and one primary caregiver. Recruitment did not depend on prior exposure to the videos later included in the study. |
| Contributor selection | Ten adult family contributors were recruited by convenience sampling and approached individually during pre-anaesthesia assessment. The group included varied caregiver roles but was not intended to be statistically representative; no qualitative saturation criterion was applied. |
| Invitation approach | Family contributors were approached individually during pre-anaesthesia assessment before their children’s scheduled procedures. Invitations were made after routine clinical communication had been completed or at another time that did not interfere with care. Participation was voluntary, and refusal did not affect the child’s medical care. |
| Format of involvement | The activity consisted of brief one-to-one, face-to-face consultations. It was an advisory involvement activity rather than a focus group, questionnaire survey or qualitative interview study. Contributors did not review, score or comment on individual short videos. |
| Topics discussed | Contributors advised the research team on information sources relevant to study framing and on understandable lay wording for concerns about paediatric anaesthesia and child neurodevelopment. They were not asked for detailed accounts of personal experiences, anxiety, consent or treatment decisions. |
| Data handling | No personal identifiers, detailed clinical information or identifiable quotations were collected. The research team kept only anonymised, aggregate consultation notes. Contributors’ views were used as advisory input and were not treated as individual-level research data. |
| Data summary and analysis | Consultation notes were summarised only to document the advice received and the research decisions it informed. No formal qualitative coding, thematic analysis, statistical analysis or saturation assessment was undertaken. The notes were not used to produce independent empirical findings about caregivers. |
| Influence on study design and interpretation | Contributors’ advice supported the use of both professional terminology and a lay question-based search phrase. It also helped the research team consider the parent-facing relevance of omissions and false reassurance when interpreting the video findings. |
| Boundary between PPIE and research data | The PPIE component was an advisory consultation within the NIHR public-involvement framework. Contributors’ views informed research decisions but were not used as data to answer a research question about caregiver perceptions or behaviour. The consultation does not establish whether or to what extent short videos influence caregiver understanding, anxiety, trust, consent decisions or treatment choices. |
| Recognition of public contributors | The manuscript recognises the family contributors’ advisory role in the Acknowledgements and Patient or Public Contribution statements. Individual names are not reported. |
| Limitations of PPIE | The consultation involved brief one-to-one discussions with 10 contributors recruited by convenience sampling from a single pre-anaesthesia assessment setting at one time point. The input was limited and may not reflect the full range of caregiver views. The consultation did not measure actual short-video exposure, caregiver anxiety, decision outcomes or downstream effects of specific videos. |

Supplementary Table S2. Dunn post hoc pairwise comparisons across uploader categories.

| **Outcome** | **Pairwise comparison** | **Dunn Z** | **Raw P** | **BH-adjusted P** | **Effect size r** | **Significant after correction** |
| --- | --- | --- | --- | --- | --- | --- |
| Followers | Anaesthesiologist - Hospital | -0.2275 | 0.82 | 1 | 0.023 | No |
| Followers | Anaesthesiologist - News agencies | -0.1748 | 0.8613 | 1 | 0.018 | No |
| Followers | Anaesthesiologist - Non-anaesthesiologist | -0.4917 | 0.6229 | 1 | 0.050 | No |
| Followers | Anaesthesiologist - Science communicators | 1.7146 | 0.0864 | 0.2881 | 0.176 | No |
| Followers | Hospital - News agencies | 0.0074 | 0.9941 | 0.9941 | 0.001 | No |
| Followers | Hospital - Non-anaesthesiologist | -0.1575 | 0.8749 | 1 | 0.016 | No |
| Followers | Hospital - Science communicators | 1.8411 | 0.0656 | 0.328 | 0.189 | No |
| Followers | News agencies - Non-anaesthesiologist | -0.1233 | 0.9019 | 1 | 0.013 | No |
| Followers | News agencies - Science communicators | 1.5130 | 0.1303 | 0.3257 | 0.155 | No |
| Followers | Non-anaesthesiologist - Science communicators | 2.5454 | 0.0109 | 0.1091 | 0.261 | No |
| Likes | Anaesthesiologist - Hospital | 1.7618 | 0.0781 | 0.1952 | 0.181 | No |
| Likes | Anaesthesiologist - News agencies | 2.0833 | 0.0372 | 0.1241 | 0.214 | No |
| Likes | Anaesthesiologist - Non-anaesthesiologist | 1.2878 | 0.1978 | 0.2826 | 0.132 | No |
| Likes | Anaesthesiologist - Science communicators | 3.1069 | 0.0019 | 0.0189 | 0.319 | Yes |
| Likes | Hospital - News agencies | 0.6479 | 0.517 | 0.5745 | 0.066 | No |
| Likes | Hospital - Non-anaesthesiologist | -1.0650 | 0.2869 | 0.3586 | 0.109 | No |
| Likes | Hospital - Science communicators | 1.3436 | 0.1791 | 0.2984 | 0.138 | No |
| Likes | News agencies - Non-anaesthesiologist | -1.5448 | 0.1224 | 0.2448 | 0.158 | No |
| Likes | News agencies - Science communicators | 0.4764 | 0.6338 | 0.6338 | 0.049 | No |
| Likes | Non-anaesthesiologist - Science communicators | 2.7524 | 0.0059 | 0.0296 | 0.282 | Yes |
| Comments | Anaesthesiologist - Hospital | 2.7045 | 0.0068 | 0.0342 | 0.277 | Yes |
| Comments | Anaesthesiologist - News agencies | 2.2989 | 0.0215 | 0.0538 | 0.236 | No |
| Comments | Anaesthesiologist - Non-anaesthesiologist | 1.4135 | 0.1575 | 0.225 | 0.145 | No |
| Comments | Anaesthesiologist - Science communicators | 2.9730 | 0.0029 | 0.0295 | 0.305 | Yes |
| Comments | Hospital - News agencies | 0.1260 | 0.8997 | 0.8997 | 0.013 | No |
| Comments | Hospital - Non-anaesthesiologist | -2.1455 | 0.0319 | 0.0638 | 0.220 | No |
| Comments | Hospital - Science communicators | 0.3521 | 0.7248 | 0.906 | 0.036 | No |
| Comments | News agencies - Non-anaesthesiologist | -1.7097 | 0.0873 | 0.1455 | 0.175 | No |
| Comments | News agencies - Science communicators | 0.1676 | 0.8669 | 0.9632 | 0.017 | No |
| Comments | Non-anaesthesiologist - Science communicators | 2.4791 | 0.0132 | 0.0439 | 0.254 | Yes |
| Collections | Anaesthesiologist - Hospital | 1.6820 | 0.0926 | 0.3085 | 0.173 | No |
| Collections | Anaesthesiologist - News agencies | 1.5288 | 0.1263 | 0.3158 | 0.157 | No |
| Collections | Anaesthesiologist - Non-anaesthesiologist | 0.7709 | 0.4408 | 0.551 | 0.079 | No |
| Collections | Anaesthesiologist - Science communicators | 2.6387 | 0.0083 | 0.0832 | 0.271 | No |
| Collections | Hospital - News agencies | 0.1741 | 0.8618 | 0.8618 | 0.018 | No |
| Collections | Hospital - Non-anaesthesiologist | -1.4325 | 0.152 | 0.304 | 0.147 | No |
| Collections | Hospital - Science communicators | 0.9709 | 0.3316 | 0.4737 | 0.100 | No |
| Collections | News agencies - Non-anaesthesiologist | -1.2484 | 0.2119 | 0.3531 | 0.128 | No |
| Collections | News agencies - Science communicators | 0.6317 | 0.5276 | 0.5862 | 0.065 | No |
| Collections | Non-anaesthesiologist - Science communicators | 2.6133 | 0.009 | 0.0448 | 0.268 | Yes |
| Shares | Anaesthesiologist - Hospital | 0.8282 | 0.4076 | 0.6793 | 0.085 | No |
| Shares | Anaesthesiologist - News agencies | 1.3848 | 0.1661 | 0.4153 | 0.142 | No |
| Shares | Anaesthesiologist - Non-anaesthesiologist | 0.2644 | 0.7914 | 0.7914 | 0.027 | No |
| Shares | Anaesthesiologist - Science communicators | 2.1187 | 0.0341 | 0.1706 | 0.217 | No |
| Shares | Hospital - News agencies | 0.6963 | 0.4862 | 0.6078 | 0.071 | No |
| Shares | Hospital - Non-anaesthesiologist | -0.8096 | 0.4182 | 0.5974 | 0.083 | No |
| Shares | Hospital - Science communicators | 1.2584 | 0.2083 | 0.4165 | 0.129 | No |
| Shares | News agencies - Non-anaesthesiologist | -1.4167 | 0.1566 | 0.5219 | 0.145 | No |
| Shares | News agencies - Science communicators | 0.3588 | 0.7198 | 0.7997 | 0.037 | No |
| Shares | Non-anaesthesiologist - Science communicators | 2.4009 | 0.0164 | 0.1635 | 0.246 | No |
| Days since published | Anaesthesiologist - Hospital | 0.3766 | 0.7064 | 1 | 0.039 | No |
| Days since published | Anaesthesiologist - News agencies | 0.0950 | 0.9243 | 0.9243 | 0.010 | No |
| Days since published | Anaesthesiologist - Non-anaesthesiologist | 1.5772 | 0.1147 | 1 | 0.162 | No |
| Days since published | Anaesthesiologist - Science communicators | -0.1803 | 0.8569 | 0.9521 | 0.018 | No |
| Days since published | Hospital - News agencies | -0.1999 | 0.8415 | 1 | 0.021 | No |
| Days since published | Hospital - Non-anaesthesiologist | 0.9524 | 0.3409 | 1 | 0.098 | No |
| Days since published | Hospital - Science communicators | -0.5169 | 0.6052 | 1 | 0.053 | No |
| Days since published | News agencies - Non-anaesthesiologist | 0.9302 | 0.3523 | 0.8807 | 0.095 | No |
| Days since published | News agencies - Science communicators | -0.2315 | 0.8169 | 1 | 0.024 | No |
| Days since published | Non-anaesthesiologist - Science communicators | -1.5700 | 0.1164 | 0.582 | 0.161 | No |
| Duration | Anaesthesiologist - Hospital | 1.6695 | 0.095 | 0.19 | 0.171 | No |
| Duration | Anaesthesiologist - News agencies | -0.9384 | 0.348 | 0.4351 | 0.096 | No |
| Duration | Anaesthesiologist - Non-anaesthesiologist | 2.9424 | 0.0033 | 0.0163 | 0.302 | Yes |
| Duration | Anaesthesiologist - Science communicators | 0.3881 | 0.6979 | 0.6979 | 0.040 | No |
| Duration | Hospital - News agencies | -2.1997 | 0.0278 | 0.0927 | 0.226 | No |
| Duration | Hospital - Non-anaesthesiologist | 0.5518 | 0.5811 | 0.6457 | 0.057 | No |
| Duration | Hospital - Science communicators | -1.1605 | 0.2458 | 0.3512 | 0.119 | No |
| Duration | News agencies - Non-anaesthesiologist | 3.0172 | 0.0026 | 0.0255 | 0.310 | Yes |
| Duration | News agencies - Science communicators | 1.1909 | 0.2337 | 0.3895 | 0.122 | No |
| Duration | Non-anaesthesiologist - Science communicators | -2.0307 | 0.0423 | 0.1057 | 0.208 | No |
| mDISCERN | Anaesthesiologist - Hospital | 0.8078 | 0.4192 | 0.5989 | 0.083 | No |
| mDISCERN | Anaesthesiologist - News agencies | -0.0917 | 0.9269 | 0.9269 | 0.009 | No |
| mDISCERN | Anaesthesiologist - Non-anaesthesiologist | 3.5910 | <0.001 | 0.0033 | 0.368 | Yes |
| mDISCERN | Anaesthesiologist - Science communicators | 2.1955 | 0.0281 | 0.0703 | 0.225 | No |
| mDISCERN | Hospital - News agencies | -0.7143 | 0.475 | 0.5938 | 0.073 | No |
| mDISCERN | Hospital - Non-anaesthesiologist | 2.2315 | 0.0256 | 0.0855 | 0.229 | No |
| mDISCERN | Hospital - Science communicators | 1.3501 | 0.177 | 0.295 | 0.139 | No |
| mDISCERN | News agencies - Non-anaesthesiologist | 2.4719 | 0.0134 | 0.0672 | 0.254 | No |
| mDISCERN | News agencies - Science communicators | 1.8128 | 0.0699 | 0.1397 | 0.186 | No |
| mDISCERN | Non-anaesthesiologist - Science communicators | -0.3437 | 0.7311 | 0.8123 | 0.035 | No |
| MQ-VET | Anaesthesiologist - Hospital | 0.4689 | 0.6391 | 0.7989 | 0.048 | No |
| MQ-VET | Anaesthesiologist - News agencies | -0.0767 | 0.9388 | 0.9388 | 0.008 | No |
| MQ-VET | Anaesthesiologist - Non-anaesthesiologist | 3.8679 | <0.001 | 0.0011 | 0.397 | Yes |
| MQ-VET | Anaesthesiologist - Science communicators | 3.4265 | <0.001 | 0.0031 | 0.352 | Yes |
| MQ-VET | Hospital - News agencies | -0.4373 | 0.6619 | 0.7354 | 0.045 | No |
| MQ-VET | Hospital - Non-anaesthesiologist | 2.9119 | 0.0036 | 0.012 | 0.299 | Yes |
| MQ-VET | Hospital - Science communicators | 2.8330 | 0.0046 | 0.0115 | 0.291 | Yes |
| MQ-VET | News agencies - Non-anaesthesiologist | 2.6371 | 0.0084 | 0.0139 | 0.271 | Yes |
| MQ-VET | News agencies - Science communicators | 2.7665 | 0.0057 | 0.0113 | 0.284 | Yes |
| MQ-VET | Non-anaesthesiologist - Science communicators | 0.9461 | 0.3441 | 0.4916 | 0.097 | No |
| GQS | Anaesthesiologist - Hospital | -0.2261 | 0.8211 | 0.9123 | 0.023 | No |
| GQS | Anaesthesiologist - News agencies | -0.2931 | 0.7695 | 0.9619 | 0.030 | No |
| GQS | Anaesthesiologist - Non-anaesthesiologist | 3.2393 | 0.0012 | 0.012 | 0.332 | Yes |
| GQS | Anaesthesiologist - Science communicators | 2.5629 | 0.0104 | 0.026 | 0.263 | Yes |
| GQS | Hospital - News agencies | -0.1080 | 0.914 | 0.914 | 0.011 | No |
| GQS | Hospital - Non-anaesthesiologist | 3.2228 | 0.0013 | 0.0063 | 0.331 | Yes |
| GQS | Hospital - Science communicators | 2.6476 | 0.0081 | 0.027 | 0.272 | Yes |
| GQS | News agencies - Non-anaesthesiologist | 2.4714 | 0.0135 | 0.0269 | 0.254 | Yes |
| GQS | News agencies - Science communicators | 2.2917 | 0.0219 | 0.0365 | 0.235 | Yes |
| GQS | Non-anaesthesiologist - Science communicators | 0.4120 | 0.6804 | 0.9719 | 0.042 | No |
| JAMA | Anaesthesiologist - Hospital | 0.0358 | 0.9714 | 0.9714 | 0.004 | No |
| JAMA | Anaesthesiologist - News agencies | -0.3850 | 0.7002 | 0.778 | 0.040 | No |
| JAMA | Anaesthesiologist - Non-anaesthesiologist | 3.0366 | 0.0024 | 0.0239 | 0.312 | Yes |
| JAMA | Anaesthesiologist - Science communicators | 2.5758 | 0.01 | 0.0333 | 0.264 | Yes |
| JAMA | Hospital - News agencies | -0.3997 | 0.6894 | 0.8617 | 0.041 | No |
| JAMA | Hospital - Non-anaesthesiologist | 2.7071 | 0.0068 | 0.0339 | 0.278 | Yes |
| JAMA | Hospital - Science communicators | 2.4199 | 0.0155 | 0.0311 | 0.248 | Yes |
| JAMA | News agencies - Non-anaesthesiologist | 2.4435 | 0.0145 | 0.0364 | 0.251 | Yes |
| JAMA | News agencies - Science communicators | 2.3887 | 0.0169 | 0.0282 | 0.245 | Yes |
| JAMA | Non-anaesthesiologist - Science communicators | 0.6011 | 0.5477 | 0.7825 | 0.062 | No |
| PEMAT Understandability | Anaesthesiologist - Hospital | 0.7171 | 0.4733 | 0.6762 | 0.074 | No |
| PEMAT Understandability | Anaesthesiologist - News agencies | 0.2324 | 0.8162 | 0.9069 | 0.024 | No |
| PEMAT Understandability | Anaesthesiologist - Non-anaesthesiologist | 3.0779 | 0.0021 | 0.0208 | 0.316 | Yes |
| PEMAT Understandability | Anaesthesiologist - Science communicators | 2.2920 | 0.0219 | 0.1095 | 0.235 | No |
| PEMAT Understandability | Hospital - News agencies | -0.3309 | 0.7407 | 0.9259 | 0.034 | No |
| PEMAT Understandability | Hospital - Non-anaesthesiologist | 1.8814 | 0.0599 | 0.1997 | 0.193 | No |
| PEMAT Understandability | Hospital - Science communicators | 1.5252 | 0.1272 | 0.212 | 0.156 | No |
| PEMAT Understandability | News agencies - Non-anaesthesiologist | 1.7613 | 0.0782 | 0.1955 | 0.181 | No |
| PEMAT Understandability | News agencies - Science communicators | 1.5827 | 0.1135 | 0.227 | 0.162 | No |
| PEMAT Understandability | Non-anaesthesiologist - Science communicators | 0.2139 | 0.8306 | 0.8306 | 0.022 | No |
| PEMAT Actionability | Anaesthesiologist - Hospital | 0.3676 | 0.7132 | 0.7924 | 0.038 | No |
| PEMAT Actionability | Anaesthesiologist - News agencies | -0.6991 | 0.4845 | 0.6056 | 0.072 | No |
| PEMAT Actionability | Anaesthesiologist - Non-anaesthesiologist | 2.1205 | 0.034 | 0.1698 | 0.218 | No |
| PEMAT Actionability | Anaesthesiologist - Science communicators | 1.7172 | 0.0859 | 0.2148 | 0.176 | No |
| PEMAT Actionability | Hospital - News agencies | -0.9601 | 0.337 | 0.4815 | 0.099 | No |
| PEMAT Actionability | Hospital - Non-anaesthesiologist | 1.4564 | 0.1453 | 0.2906 | 0.149 | No |
| PEMAT Actionability | Hospital - Science communicators | 1.2983 | 0.1942 | 0.3237 | 0.133 | No |
| PEMAT Actionability | News agencies - Non-anaesthesiologist | 2.2006 | 0.0278 | 0.2776 | 0.226 | No |
| PEMAT Actionability | News agencies - Science communicators | 2.0100 | 0.0444 | 0.1481 | 0.206 | No |
| PEMAT Actionability | Non-anaesthesiologist - Science communicators | 0.3187 | 0.75 | 0.75 | 0.033 | No |

Note. Dunn post hoc tests followed Kruskal-Wallis tests for uploader-category comparisons. P values were adjusted with the Benjamini-Hochberg method. Pairwise effect size r was calculated as |Z|/√N, with N = 95. Results should be interpreted cautiously for small uploader categories, particularly news agencies and science communicators.

Supplementary Table S3. Stratified misinformation characteristics.

| **Stratification** | **Subgroup** | **n** | **Any misinformation, n/N (%)** | **False safety assurances, n/N (%)** | **Overgeneralisation, n/N (%)** | **Exaggeration/absolute claims, n/N (%)** |
| --- | --- | --- | --- | --- | --- | --- |
| Platform | Bilibili | 21 | 10/21 (47.6) | 8/21 (38.1) | 2/21 (9.5) | 0/21 (0.0) |
| Platform | Douyin | 74 | 41/74 (55.4) | 35/74 (47.3) | 3/74 (4.1) | 3/74 (4.1) |
| Uploader type | Clinical or hospital sources | 84 | 43/84 (51.2) | 37/84 (44.0) | 3/84 (3.6) | 3/84 (3.6) |
| Uploader type | Non-medical sources | 11 | 8/11 (72.7) | 6/11 (54.5) | 2/11 (18.2) | 0/11 (0.0) |
| Uploader category | Anaesthesiologist | 10 | 3/10 (30.0) | 2/10 (20.0) | 1/10 (10.0) | 0/10 (0.0) |
| Uploader category | Non-anaesthesiologist | 66 | 35/66 (53.0) | 30/66 (45.5) | 2/66 (3.0) | 3/66 (4.5) |
| Uploader category | Hospital | 8 | 5/8 (62.5) | 5/8 (62.5) | 0/8 (0.0) | 0/8 (0.0) |
| Uploader category | News agencies | 4 | 1/4 (25.0) | 1/4 (25.0) | 0/4 (0.0) | 0/4 (0.0) |
| Uploader category | Science communicators | 7 | 7/7 (100.0) | 5/7 (71.4) | 2/7 (28.6) | 0/7 (0.0) |

Note. Values are presented as n/N (%). Any misinformation indicates the presence of at least one predefined misinformation category.

Supplementary Table S4. Exact tests for subgroup differences in misinformation outcomes.

| **Stratification** | **Outcome** | **Test** | **Raw P value** | **BH-adjusted P value** | **Cramer's V** | **Effect size magnitude** | **Yes counts** |
| --- | --- | --- | --- | --- | --- | --- | --- |
| Platform | Any misinformation | Fisher exact test | 0.623 | 0.830 | 0.065 | negligible | Bilibili: 10/21; Douyin: 41/74 |
| Platform | False safety assurances | Fisher exact test | 0.620 | 0.830 | 0.077 | negligible | Bilibili: 8/21; Douyin: 35/74 |
| Platform | Overgeneralisation | Fisher exact test | 0.305 | 0.610 | 0.102 | small | Bilibili: 2/21; Douyin: 3/74 |
| Platform | Exaggeration/absolute claims | Fisher exact test | 1.000 | 1.000 | 0.096 | negligible | Bilibili: 0/21; Douyin: 3/74 |
| Uploader type | Any misinformation | Fisher exact test | 0.213 | 0.512 | 0.138 | small | Clinical or hospital sources: 43/84; Non-medical sources: 8/11 |
| Uploader type | False safety assurances | Fisher exact test | 0.538 | 0.830 | 0.067 | negligible | Clinical or hospital sources: 37/84; Non-medical sources: 6/11 |
| Uploader type | Overgeneralisation | Fisher exact test | 0.101 | 0.403 | 0.209 | small | Clinical or hospital sources: 3/84; Non-medical sources: 2/11 |
| Uploader type | Exaggeration/absolute claims | Fisher exact test | 1.000 | 1.000 | 0.065 | negligible | Clinical or hospital sources: 3/84; Non-medical sources: 0/11 |
| Uploader category | Any misinformation | Fisher-Freeman-Halton exact test | 0.030 | 0.355 | 0.322 | moderate | Anaesthesiologist: 3/10; Non-anaesthesiologist: 35/66; Hospital: 5/8; News agencies: 1/4; Science communicators: 7/7 |
| Uploader category | False safety assurances | Fisher-Freeman-Halton exact test | 0.198 | 0.512 | 0.254 | small | Anaesthesiologist: 2/10; Non-anaesthesiologist: 30/66; Hospital: 5/8; News agencies: 1/4; Science communicators: 5/7 |
| Uploader category | Overgeneralisation | Fisher-Freeman-Halton exact test | 0.083 | 0.403 | 0.315 | moderate | Anaesthesiologist: 1/10; Non-anaesthesiologist: 2/66; Hospital: 0/8; News agencies: 0/4; Science communicators: 2/7 |
| Uploader category | Exaggeration/absolute claims | Fisher-Freeman-Halton exact test | 1.000 | 1.000 | 0.120 | small | Anaesthesiologist: 0/10; Non-anaesthesiologist: 3/66; Hospital: 0/8; News agencies: 0/4; Science communicators: 0/7 |

Note. Two-group comparisons were performed using Fisher's exact test. Uploader-category comparisons were performed using the Fisher-Freeman-Halton exact test. P values were adjusted using the Benjamini-Hochberg method across the stratified misinformation tests. Cramer's V was reported as the effect size.

Supplementary Table S5. Spearman correlation analysis between video characteristics, engagement metrics, and quality-related scores.

| **Section** | **Variable 1** | **Variable 2** | **rho** | **Raw P** | **BH-adjusted P** | **Interpretation** |
| --- | --- | --- | --- | --- | --- | --- |
| Video characteristics and engagement metrics | Rank | Followers | -0.287 | 0.005 | 0.016 | weak negative; significant after BH correction |
| Video characteristics and engagement metrics | Rank | Likes | -0.225 | 0.028 | 0.070 | weak negative; not significant after BH correction |
| Video characteristics and engagement metrics | Rank | Comments | -0.154 | 0.137 | 0.248 | weak negative; not significant after BH correction |
| Video characteristics and engagement metrics | Rank | Collections | -0.258 | 0.012 | 0.031 | weak negative; significant after BH correction |
| Video characteristics and engagement metrics | Rank | Shares | -0.201 | 0.050 | 0.108 | weak negative; not significant after BH correction |
| Video characteristics intercorrelations | Rank | Days since published | -0.057 | 0.586 | 0.698 | negligible negative; not significant after BH correction |
| Video characteristics intercorrelations | Rank | Duration | -0.150 | 0.146 | 0.257 | weak negative; not significant after BH correction |
| Video characteristics and quality scores | Rank | mDISCERN | -0.064 | 0.536 | 0.670 | negligible negative; not significant after BH correction |
| Video characteristics and quality scores | Rank | GQS | 0.039 | 0.711 | 0.815 | negligible positive; not significant after BH correction |
| Video characteristics and quality scores | Rank | MQ-VET | 0.009 | 0.935 | 0.963 | negligible positive; not significant after BH correction |
| Video characteristics and quality scores | Rank | JAMA | -0.057 | 0.586 | 0.698 | negligible negative; not significant after BH correction |
| Video characteristics and quality scores | Rank | PEMAT Understandability | -0.156 | 0.130 | 0.244 | weak negative; not significant after BH correction |
| Video characteristics and quality scores | Rank | PEMAT Actionability | 0.121 | 0.243 | 0.388 | weak positive; not significant after BH correction |
| Engagement metrics intercorrelations | Followers | Likes | 0.691 | <0.001 | <0.001 | strong positive; significant after BH correction |
| Engagement metrics intercorrelations | Followers | Comments | 0.601 | <0.001 | <0.001 | strong positive; significant after BH correction |
| Engagement metrics intercorrelations | Followers | Collections | 0.575 | <0.001 | <0.001 | strong positive; significant after BH correction |
| Engagement metrics intercorrelations | Followers | Shares | 0.500 | <0.001 | <0.001 | strong positive; significant after BH correction |
| Video characteristics and engagement metrics | Followers | Days since published | 0.084 | 0.419 | 0.577 | negligible positive; not significant after BH correction |
| Video characteristics and engagement metrics | Followers | Duration | -0.068 | 0.514 | 0.648 | negligible negative; not significant after BH correction |
| Engagement metrics and quality scores | Followers | mDISCERN | -0.140 | 0.175 | 0.297 | weak negative; not significant after BH correction |
| Engagement metrics and quality scores | Followers | GQS | -0.103 | 0.323 | 0.486 | weak negative; not significant after BH correction |
| Engagement metrics and quality scores | Followers | MQ-VET | -0.156 | 0.131 | 0.244 | weak negative; not significant after BH correction |
| Engagement metrics and quality scores | Followers | JAMA | -0.277 | 0.007 | 0.020 | weak negative; significant after BH correction |
| Engagement metrics and quality scores | Followers | PEMAT Understandability | -0.037 | 0.719 | 0.821 | negligible negative; not significant after BH correction |
| Engagement metrics and quality scores | Followers | PEMAT Actionability | -0.159 | 0.123 | 0.244 | weak negative; not significant after BH correction |
| Engagement metrics intercorrelations | Likes | Comments | 0.891 | <0.001 | <0.001 | strong positive; significant after BH correction |
| Engagement metrics intercorrelations | Likes | Collections | 0.892 | <0.001 | <0.001 | strong positive; significant after BH correction |
| Engagement metrics intercorrelations | Likes | Shares | 0.814 | <0.001 | <0.001 | strong positive; significant after BH correction |
| Video characteristics and engagement metrics | Likes | Days since published | 0.212 | 0.039 | 0.087 | weak positive; not significant after BH correction |
| Video characteristics and engagement metrics | Likes | Duration | -0.152 | 0.142 | 0.252 | weak negative; not significant after BH correction |
| Engagement metrics and quality scores | Likes | mDISCERN | -0.070 | 0.503 | 0.641 | negligible negative; not significant after BH correction |
| Engagement metrics and quality scores | Likes | GQS | 0.000 | 0.999 | 1.000 | No correlation; not significant after BH correction |
| Engagement metrics and quality scores | Likes | MQ-VET | -0.060 | 0.564 | 0.693 | negligible negative; not significant after BH correction |
| Engagement metrics and quality scores | Likes | JAMA | -0.217 | 0.035 | 0.079 | weak negative; not significant after BH correction |
| Engagement metrics and quality scores | Likes | PEMAT Understandability | 0.055 | 0.599 | 0.704 | negligible positive; not significant after BH correction |
| Engagement metrics and quality scores | Likes | PEMAT Actionability | 0.011 | 0.917 | 0.963 | negligible positive; not significant after BH correction |
| Engagement metrics intercorrelations | Comments | Collections | 0.806 | <0.001 | <0.001 | strong positive; significant after BH correction |
| Engagement metrics intercorrelations | Comments | Shares | 0.787 | <0.001 | <0.001 | strong positive; significant after BH correction |
| Video characteristics and engagement metrics | Comments | Days since published | 0.229 | 0.026 | 0.065 | weak positive; not significant after BH correction |
| Video characteristics and engagement metrics | Comments | Duration | -0.156 | 0.131 | 0.244 | weak negative; not significant after BH correction |
| Engagement metrics and quality scores | Comments | mDISCERN | -0.081 | 0.434 | 0.582 | negligible negative; not significant after BH correction |
| Engagement metrics and quality scores | Comments | GQS | -0.106 | 0.308 | 0.473 | weak negative; not significant after BH correction |
| Engagement metrics and quality scores | Comments | MQ-VET | -0.144 | 0.164 | 0.281 | weak negative; not significant after BH correction |
| Engagement metrics and quality scores | Comments | JAMA | -0.261 | 0.011 | 0.029 | weak negative; significant after BH correction |
| Engagement metrics and quality scores | Comments | PEMAT Understandability | -0.076 | 0.465 | 0.603 | negligible negative; not significant after BH correction |
| Engagement metrics and quality scores | Comments | PEMAT Actionability | -0.009 | 0.930 | 0.963 | negligible negative; not significant after BH correction |
| Engagement metrics intercorrelations | Collections | Shares | 0.908 | <0.001 | <0.001 | strong positive; significant after BH correction |
| Video characteristics and engagement metrics | Collections | Days since published | 0.158 | 0.125 | 0.244 | weak positive; not significant after BH correction |
| Video characteristics and engagement metrics | Collections | Duration | -0.134 | 0.195 | 0.323 | weak negative; not significant after BH correction |
| Engagement metrics and quality scores | Collections | mDISCERN | -0.091 | 0.382 | 0.550 | negligible negative; not significant after BH correction |
| Engagement metrics and quality scores | Collections | GQS | -0.010 | 0.923 | 0.963 | negligible negative; not significant after BH correction |
| Engagement metrics and quality scores | Collections | MQ-VET | -0.089 | 0.390 | 0.556 | negligible negative; not significant after BH correction |
| Engagement metrics and quality scores | Collections | JAMA | -0.217 | 0.035 | 0.079 | weak negative; not significant after BH correction |
| Engagement metrics and quality scores | Collections | PEMAT Understandability | 0.087 | 0.402 | 0.563 | negligible positive; not significant after BH correction |
| Engagement metrics and quality scores | Collections | PEMAT Actionability | 0.003 | 0.980 | 0.988 | negligible positive; not significant after BH correction |
| Video characteristics and engagement metrics | Shares | Days since published | 0.272 | 0.008 | 0.022 | weak positive; significant after BH correction |
| Video characteristics and engagement metrics | Shares | Duration | -0.267 | 0.009 | 0.025 | weak negative; significant after BH correction |
| Engagement metrics and quality scores | Shares | mDISCERN | -0.101 | 0.331 | 0.492 | weak negative; not significant after BH correction |
| Engagement metrics and quality scores | Shares | GQS | -0.030 | 0.776 | 0.868 | negligible negative; not significant after BH correction |
| Engagement metrics and quality scores | Shares | MQ-VET | -0.132 | 0.201 | 0.329 | weak negative; not significant after BH correction |
| Engagement metrics and quality scores | Shares | JAMA | -0.222 | 0.030 | 0.073 | weak negative; not significant after BH correction |
| Engagement metrics and quality scores | Shares | PEMAT Understandability | 0.025 | 0.809 | 0.888 | negligible positive; not significant after BH correction |
| Engagement metrics and quality scores | Shares | PEMAT Actionability | 0.081 | 0.436 | 0.582 | negligible positive; not significant after BH correction |
| Video characteristics intercorrelations | Days since published | Duration | -0.115 | 0.268 | 0.419 | weak negative; not significant after BH correction |
| Video characteristics and quality scores | Days since published | mDISCERN | 0.098 | 0.347 | 0.506 | negligible positive; not significant after BH correction |
| Video characteristics and quality scores | Days since published | GQS | -0.012 | 0.905 | 0.963 | negligible negative; not significant after BH correction |
| Video characteristics and quality scores | Days since published | MQ-VET | -0.054 | 0.605 | 0.704 | negligible negative; not significant after BH correction |
| Video characteristics and quality scores | Days since published | JAMA | -0.025 | 0.813 | 0.888 | negligible negative; not significant after BH correction |
| Video characteristics and quality scores | Days since published | PEMAT Understandability | -0.078 | 0.450 | 0.597 | negligible negative; not significant after BH correction |
| Video characteristics and quality scores | Days since published | PEMAT Actionability | 0.007 | 0.947 | 0.968 | negligible positive; not significant after BH correction |
| Video characteristics and quality scores | Duration | mDISCERN | 0.335 | 0.001 | 0.003 | moderate positive; significant after BH correction |
| Video characteristics and quality scores | Duration | GQS | 0.381 | <0.001 | <0.001 | moderate positive; significant after BH correction |
| Video characteristics and quality scores | Duration | MQ-VET | 0.285 | 0.005 | 0.016 | weak positive; significant after BH correction |
| Video characteristics and quality scores | Duration | JAMA | 0.208 | 0.043 | 0.093 | weak positive; not significant after BH correction |
| Video characteristics and quality scores | Duration | PEMAT Understandability | 0.238 | 0.020 | 0.053 | weak positive; not significant after BH correction |
| Video characteristics and quality scores | Duration | PEMAT Actionability | 0.194 | 0.059 | 0.123 | weak positive; not significant after BH correction |
| Quality-score intercorrelations | mDISCERN | GQS | 0.680 | <0.001 | <0.001 | strong positive; significant after BH correction |
| Quality-score intercorrelations | mDISCERN | MQ-VET | 0.706 | <0.001 | <0.001 | strong positive; significant after BH correction |
| Quality-score intercorrelations | mDISCERN | JAMA | 0.471 | <0.001 | <0.001 | moderate positive; significant after BH correction |
| Quality-score intercorrelations | mDISCERN | PEMAT Understandability | 0.522 | <0.001 | <0.001 | strong positive; significant after BH correction |
| Quality-score intercorrelations | mDISCERN | PEMAT Actionability | 0.468 | <0.001 | <0.001 | moderate positive; significant after BH correction |
| Quality-score intercorrelations | GQS | MQ-VET | 0.816 | <0.001 | <0.001 | strong positive; significant after BH correction |
| Quality-score intercorrelations | GQS | JAMA | 0.544 | <0.001 | <0.001 | strong positive; significant after BH correction |
| Quality-score intercorrelations | GQS | PEMAT Understandability | 0.683 | <0.001 | <0.001 | strong positive; significant after BH correction |
| Quality-score intercorrelations | GQS | PEMAT Actionability | 0.629 | <0.001 | <0.001 | strong positive; significant after BH correction |
| Quality-score intercorrelations | MQ-VET | JAMA | 0.704 | <0.001 | <0.001 | strong positive; significant after BH correction |
| Quality-score intercorrelations | MQ-VET | PEMAT Understandability | 0.675 | <0.001 | <0.001 | strong positive; significant after BH correction |
| Quality-score intercorrelations | MQ-VET | PEMAT Actionability | 0.642 | <0.001 | <0.001 | strong positive; significant after BH correction |
| Quality-score intercorrelations | JAMA | PEMAT Understandability | 0.425 | <0.001 | <0.001 | moderate positive; significant after BH correction |
| Quality-score intercorrelations | JAMA | PEMAT Actionability | 0.401 | <0.001 | <0.001 | moderate positive; significant after BH correction |
| Quality-score intercorrelations | PEMAT Understandability | PEMAT Actionability | 0.513 | <0.001 | <0.001 | strong positive; significant after BH correction |

Note. Spearman correlation coefficients are reported as rho. Raw P values and Benjamini-Hochberg-adjusted P values are shown for 91 unique pairwise correlations. Values reported as <0.001 were below 0.001 after rounding. Adjusted P values <0.05 were considered significant.

Supplementary Table S6. Platform-balanced resampling sensitivity analysis.

| **Variable** | **Direction** | **Original P** | **Original r** | **Median P** | **P IQR** | **Median r** | **Same direction %** | **Significant repeats %** |
| --- | --- | --- | --- | --- | --- | --- | --- | --- |
| Followers | Douyin > Bilibili | 0.012 | 0.259 | 0.047 | 0.030–0.074 | 0.309 | 100.0 | 52.6 |
| Likes | Douyin > Bilibili | <0.001 | 0.358 | 0.006 | 0.004–0.009 | 0.429 | 100.0 | 100.0 |
| Comments | Douyin > Bilibili | 0.002 | 0.321 | 0.012 | 0.007–0.022 | 0.388 | 100.0 | 94.5 |
| Collections | Douyin > Bilibili | <0.001 | 0.389 | 0.003 | 0.001–0.005 | 0.464 | 100.0 | 100.0 |
| Shares | Douyin > Bilibili | <0.001 | 0.481 | <0.001 | <0.001 | 0.578 | 100.0 | 100.0 |
| Days since published | Bilibili > Douyin | 0.179 | 0.139 | 0.268 | 0.145–0.450 | 0.173 | 88.5 | 4.9 |
| Duration | Bilibili > Douyin | 0.001 | 0.331 | 0.010 | 0.004–0.029 | 0.398 | 100.0 | 86.9 |
| mDISCERN | Equal medians | 0.473 | 0.074 | 0.546 | 0.363–0.755 | 0.095 | 73.4 | 0.3 |
| MQ-VET | Bilibili > Douyin | 0.964 | 0.004 | 0.772 | 0.641–0.900 | 0.047 | 68.8 | 0.0 |
| PEMAT Understandability | Douyin > Bilibili | 0.939 | 0.008 | 0.723 | 0.561–0.880 | 0.057 | 93.1 | 0.0 |
| PEMAT Actionability | Equal medians | 0.277 | 0.112 | 0.428 | 0.239–0.655 | 0.124 | 99.8 | 1.9 |
| GQS | Equal medians | 0.731 | 0.035 | 0.721 | 0.541–0.884 | 0.057 | 99.8 | 0.2 |
| JAMA | Equal medians | 0.748 | 0.033 | 0.692 | 0.490–0.857 | 0.063 | 93.8 | 0.0 |

Supplementary Table S7. Collapsed uploader-category sensitivity analyses.

| **Analysis** | **Variable** | **Group 1, M (Q1, Q3)** | **Group 2, M (Q1, Q3)** | **Z** | **P** | **Effect size r** | **Magnitude** |
| --- | --- | --- | --- | --- | --- | --- | --- |
| Anaesthesiologist + Hospital vs Other | mDISCERN | 4.00 (3.00, 4.75) | 2.00 (2.00, 3.00) | 3.704 | <0.001 | 0.380 | medium |
| Anaesthesiologist + Hospital vs Other | MQ-VET | 47.00 (46.00, 57.25) | 38.00 (34.00, 43.00) | 4.497 | <0.001 | 0.461 | medium |
| Anaesthesiologist + Hospital vs Other | PEMAT Understandability | 78.00 (77.25, 82.75) | 75.00 (63.00, 80.00) | 3.262 | 0.001 | 0.335 | medium |
| Anaesthesiologist + Hospital vs Other | PEMAT Actionability | 67.00 (67.00, 93.75) | 67.00 (67.00, 67.00) | 2.272 | 0.023 | 0.233 | small |
| Anaesthesiologist + Hospital vs Other | GQS | 4.00 (3.00, 4.00) | 3.00 (2.00, 3.00) | 4.185 | <0.001 | 0.429 | medium |
| Anaesthesiologist + Hospital vs Other | JAMA | 1.00 (1.00, 2.00) | 1.00 (0.00, 1.00) | 3.740 | <0.001 | 0.384 | medium |
| Anaesthesiologist + Hospital + News vs Non-specialist | mDISCERN | 4.00 (3.00, 5.00) | 2.00 (2.00, 3.00) | 4.424 | <0.001 | 0.454 | medium |
| Anaesthesiologist + Hospital + News vs Non-specialist | MQ-VET | 48.50 (46.00, 57.75) | 37.00 (33.00, 42.00) | 5.246 | <0.001 | 0.538 | large |
| Anaesthesiologist + Hospital + News vs Non-specialist | PEMAT Understandability | 78.00 (77.25, 82.00) | 70.00 (63.00, 78.00) | 3.717 | <0.001 | 0.381 | medium |
| Anaesthesiologist + Hospital + News vs Non-specialist | PEMAT Actionability | 67.00 (67.00, 93.75) | 67.00 (67.00, 67.00) | 3.057 | 0.002 | 0.314 | medium |
| Anaesthesiologist + Hospital + News vs Non-specialist | GQS | 4.00 (3.00, 4.00) | 3.00 (2.00, 3.00) | 4.874 | <0.001 | 0.500 | large |
| Anaesthesiologist + Hospital + News vs Non-specialist | JAMA | 1.00 (1.00, 2.00) | 1.00 (0.00, 1.00) | 4.476 | <0.001 | 0.459 | medium |

Supplementary Table S8. Log-transformed Spearman correlation sensitivity analysis.

| **Association** | **Family** | **Original rho** | **Original adj. P** | **Log rho** | **Log adj. P** | **Interpretation** |
| --- | --- | --- | --- | --- | --- | --- |
| Followers vs mDISCERN | Engagement vs quality | -0.140 | 0.420 | -0.140 | 0.420 | Stable |
| Followers vs MQ-VET | Engagement vs quality | -0.156 | 0.362 | -0.156 | 0.362 | Stable |
| Followers vs PEMAT Understandability | Engagement vs quality | -0.037 | 0.892 | -0.037 | 0.892 | Stable |
| Followers vs PEMAT Actionability | Engagement vs quality | -0.159 | 0.362 | -0.159 | 0.362 | Stable |
| Followers vs GQS | Engagement vs quality | -0.103 | 0.627 | -0.103 | 0.627 | Stable |
| Followers vs JAMA | Engagement vs quality | -0.277 | 0.059 | -0.277 | 0.059 | Stable |
| Likes vs mDISCERN | Engagement vs quality | -0.070 | 0.696 | -0.070 | 0.696 | Stable |
| Likes vs MQ-VET | Engagement vs quality | -0.060 | 0.752 | -0.060 | 0.752 | Stable |
| Likes vs PEMAT Understandability | Engagement vs quality | 0.055 | 0.770 | 0.055 | 0.770 | Stable |
| Likes vs PEMAT Actionability | Engagement vs quality | 0.011 | 0.985 | 0.011 | 0.985 | Stable |
| Likes vs GQS | Engagement vs quality | 0.000 | 0.999 | 0.000 | 0.999 | Stable |
| Likes vs JAMA | Engagement vs quality | -0.217 | 0.140 | -0.217 | 0.140 | Stable |
| Comments vs mDISCERN | Engagement vs quality | -0.081 | 0.654 | -0.081 | 0.654 | Stable |
| Comments vs MQ-VET | Engagement vs quality | -0.144 | 0.420 | -0.144 | 0.420 | Stable |
| Comments vs PEMAT Understandability | Engagement vs quality | -0.076 | 0.669 | -0.076 | 0.669 | Stable |
| Comments vs PEMAT Actionability | Engagement vs quality | -0.009 | 0.985 | -0.009 | 0.985 | Stable |
| Comments vs GQS | Engagement vs quality | -0.106 | 0.627 | -0.106 | 0.627 | Stable |
| Comments vs JAMA | Engagement vs quality | -0.261 | 0.078 | -0.261 | 0.078 | Stable |
| Collections vs mDISCERN | Engagement vs quality | -0.091 | 0.654 | -0.091 | 0.654 | Stable |
| Collections vs MQ-VET | Engagement vs quality | -0.089 | 0.654 | -0.089 | 0.654 | Stable |
| Collections vs PEMAT Understandability | Engagement vs quality | 0.087 | 0.654 | 0.087 | 0.654 | Stable |
| Collections vs PEMAT Actionability | Engagement vs quality | 0.003 | 0.999 | 0.003 | 0.999 | Stable |
| Collections vs GQS | Engagement vs quality | -0.010 | 0.985 | -0.010 | 0.985 | Stable |
| Collections vs JAMA | Engagement vs quality | -0.217 | 0.140 | -0.217 | 0.140 | Stable |
| Shares vs mDISCERN | Engagement vs quality | -0.101 | 0.627 | -0.101 | 0.627 | Stable |
| Shares vs MQ-VET | Engagement vs quality | -0.132 | 0.453 | -0.132 | 0.453 | Stable |
| Shares vs PEMAT Understandability | Engagement vs quality | 0.025 | 0.939 | 0.025 | 0.939 | Stable |
| Shares vs PEMAT Actionability | Engagement vs quality | 0.081 | 0.654 | 0.081 | 0.654 | Stable |
| Shares vs GQS | Engagement vs quality | -0.030 | 0.931 | -0.030 | 0.931 | Stable |
| Shares vs JAMA | Engagement vs quality | -0.222 | 0.140 | -0.222 | 0.140 | Stable |
| Duration vs mDISCERN | Duration vs quality | 0.335 | 0.016 | 0.335 | 0.016 | Stable |
| Duration vs MQ-VET | Duration vs quality | 0.285 | 0.059 | 0.285 | 0.059 | Stable |
| Duration vs PEMAT Understandability | Duration vs quality | 0.238 | 0.122 | 0.238 | 0.122 | Stable |
| Duration vs PEMAT Actionability | Duration vs quality | 0.194 | 0.193 | 0.194 | 0.193 | Stable |
| Duration vs GQS | Duration vs quality | 0.381 | 0.005 | 0.381 | 0.005 | Stable |
| Duration vs JAMA | Duration vs quality | 0.208 | 0.154 | 0.208 | 0.154 | Stable |
